# Antihypertensive drug-induced liver injury is rare, but strongly associated with HLA-B∗35:01

**DOI:** 10.1016/j.apsb.2026.06.003

**Published:** 2026-06-10

**Authors:** Lily Dara, Paola Nicoletti, Chaudry Nasir Majeed, Andrew Dellinger, David E. Kleiner, Mahmoud G. Mohamed, Tatyana Kushner, Raj Vuppalanchi, Elizabeth J Phillips, David A. Ostrov, Mohammed Umair Masood, Huiman Barnhart, Jiezhun Gu, Herbert L. Bonkovsky

**Affiliations:** a Division of Gastrointestinal and Liver Diseases, Research Center for Liver Diseases, Department of Medicine, Keck School of Medicine of the University of Southern California, Los Angeles, CA 90033, USA; b Department of Genetics and Genomic Sciences, Icahn School of Medicine at Mount Sinai, New York, NY 10029, USA; c Department of Gastroenterology and Hepatology, Weill Cornell Medicine, New York, NY 10065, USA; d Department of Medicine, Vanderbilt University Medical Center, Nashville, TN 37232, USA; e Department of Pathology, Immunology and Laboratory Medicine, University of Florida College of Medicine, Gainesville, FL 32610, USA; f Division of Gastroenterology and Hepatology, Department of Medicine, Indiana University School of Medicine and Indiana University Health, Indianapolis, IN 46202, USA; g Duke Clinical Research Institute, Durham, NC 27710, USA; h Duke Molecular Physiology Institute, Duke University School of Medicine, Durham, NC 27710, USA; i Laboratory of Pathology, National Cancer Institute, Bethesda, MD 20892, USA; j Section on Gastroenterology and Hepatology, Department of Medicine, Wake Forest University School of Medicine and Advocate Health Wake Forest Baptist, Winston-Salem, NC 27101, USA

**Keywords:** Hepatotoxicity, DILI, Genetics, Hypertension, Methyldopa, Hydralazine, Lisinopril, Single nucleotide polymorphism

## Abstract

Anti-hypertensive (anti-HTN) drugs are widely prescribed, but the features of associated drug-induced liver injury (DILI) are poorly defined. A total of 2207 patients enrolled in the Drug-Induced Liver Injury Network (DILIN) from 2003 to 2024 were adjudicated as definite, highly likely, or probable DILI. Of these, 44 cases were attributed to anti-HTN drugs. These were compared with non-anti-HTN DILI cases and population controls. Human Leukocyte Antigen (HLA) sequencing was performed, and allele frequencies were analyzed using Fisher’s Exact Test. Among the 44 anti-HTN DILI cases, 14 were caused by ACE inhibitors/ARBs, 13 by methyldopa, 11 by hydralazine, and 3 each by beta-blockers and calcium-channel blockers. Methyldopa DILI presented with hepatocellular injury in young women (prescribed mainly for pregnancy-related HTN). Non-methyldopa cases were 60% hepatocellular, 28% cholestatic, and 9% mixed. Severe outcomes included two liver transplants and two deaths (lisinopril and hydralazine). HLA-B∗35:01 was markedly enriched (50% *vs.* 13% in controls; OR 6.0; *P* = 1 × 10^−8^) and was linked to shorter latency (50 *vs.* 109 days; *P* = 0.02) but not severity. HLA-B∗35:01 was overrepresented across all drug classes, including 66% of methyldopa cases, 27% of hydralazine cases, and 42% of ACE/ARB cases. DILI from anti-HTN medications is very rare but strongly associated with HLA-B∗35:01, especially for methyldopa. Carriers display a shorter latency, potentially indicating an accelerated adaptive immune response, that warrants further exploration.

## Graphical abstract

HLA-B∗35:01 is associated with susceptibility to anti-hypertensive DILI, particularly hepatocellular injury, and is linked to an accelerated immune response with shorter latency.

**Figure:**
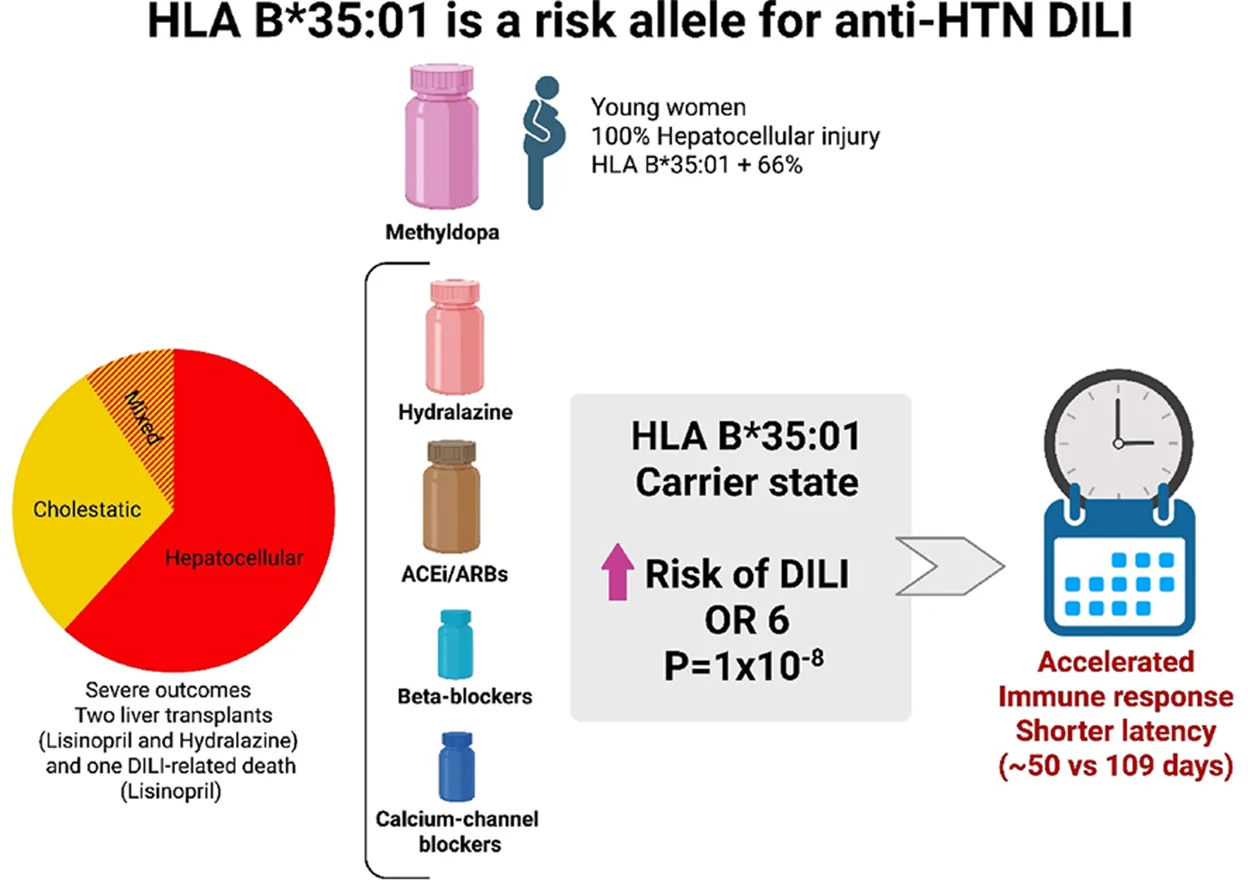

## 1 Introduction

Hypertension (HTN) is a world-wide disease with an estimated prevalence of 31% among adults and remains underdiagnosed^1^. Major classes of antihypertensives (anti-HTN) include: angiotensin converting enzyme inhibitors (ACEI), angiotensin receptor blockers (ARB), beta blockers (BB), calcium channel blockers (CCB), vasodilators, diuretics, and centrally acting antihypertensives.

Despite their overall safety, there are multiple case reports and a few case series describing drug-induced liver injury (DILI) due to anti-HTN medications with variable latency and phenotypes (summarized in Supporting Information Table S1)^2^, ^3^, ^4^, ^5^, ^6^, ^7^, ^8^, ^9^, ^10^. A recent retrospective study of 229,881 ACEI/ARB users in South Korea reported crude ACEI/ARB-related DILI ranging from 15.6 to 82.8 per 1000 person years, mostly mild and with a cholestatic pattern^11^. Case reports on DILI ascribed to Beta Blockers (BB) suggest a predominantly hepatocellular pattern of liver injury, with a latency between 30 and 210 days; and recovery within a few days to weeks after discontinuation. DILI from amlodipine, a CCB, has also been associated with hepatocellular injury, a short latency, and normalization of liver enzymes shortly after discontinuation^12^, ^13^, ^14^, ^15^, ^16^. Hydralazine and methyldopa are used to treat hypertension in pregnancy and remain on the World health Organization (WHO) list of essential drugs, as well as on the American College of Gynecology guidelines for treating HTN in pregnancy^17^, ^18^, ^19^. Both drugs have been implicated in hepatocellular injury, often presenting with drug-induced autoimmune-like hepatitis (DI-AIH), and a latency period ranging from 3 to 150 days^20^, ^21^, ^22^. Notably, hydralazine-induced liver injury can be severe, occasionally progressing to acute liver failure requiring liver transplantation^21^. Methyldopa, predominantly used during pregnancy and the peripartum period, also carries a risk of clinically significant DILI and jaundice, warranting particular attention^23^, ^24^, ^25^, ^26^, ^27^, ^28^.

Heretofore, no known genetic or acquired risk factors for developing antihypertensive DILI have been elucidated. In recent years, various groups and investigators, including prospective hepatotoxicity registries such as the U.S. Drug-Induced Liver Injury Network (DILIN), have identified multiple mechanisms underlying idiosyncratic DILI, including associations between specific single nucleotide polymorphisms (SNPs), primarily within human leukocyte antigen (HLA) genes, and liver injury caused by antibiotics, immune checkpoint inhibitors, anticonvulsants, and herbal and dietary supplements, among others^29^, ^30^, ^31^, ^32^, ^33^, ^34^, ^35^, ^36^, ^37^, ^38^, ^39^, ^40^, ^41^, ^42^, ^43^. *HLA* polymorphisms give rise to functional diversity among allotypes, affecting both peptide-binding specificity and the capacity to cross-present antigens. Among these, *HLA-B∗35:01*, a type I MHC molecule*,* is particularly notable for its ability to present peptides derived from both endogenous and exogenous sources^44^. Interestingly, *HLA-B∗35:01* has emerged as a frequent genotype that is over-represented in patients with idiosyncratic DILI due to multiple drugs and herbal supplements such as green tea extracts, *Garcinia cambogia*, and *Polygonum multiflorum*^33^^,^^39^.

We queried the DILIN database to identify cases of DILI ascribed with high confidence to anti-HTN drugs and in whom *HLA* typing had been performed. In this paper, we describe salient demographic, clinical, laboratory, and histo-pathological features of DILI due to anti-hypertensive medications, observed over the past 20 years in the US DILIN. We identify drugs that have been associated with severe DILI resulting in jaundice and liver failure and report novel genetic associations, especially high frequency of *HLA-B∗35:01,* compared with persons with non-HTN DILI and population controls.

## 2 Materials and methods

The Drug-Induced Liver Injury Network (DILIN) is a National Institutes of Health-funded prospective cohort study of idiosyncratic DILI suspected to be caused by prescription and over-the-counter medications as well as herbal and dietary supplements (HDS). The design, methods, terminology, and definitions used in the Network have been described in detail previously^38^. Briefly, consenting patients with suspected acute liver injury from drugs or herbal and dietary supplements seen at 5 to 8 US medical centers were prospectively enrolled into a registry. A central database is maintained at Duke University^45^. Clinical data, including history, labs, comorbidities, and medications, were extracted from medical records. All patients gave written informed consent, and the study was approved by institutional review boards and an NIDDK-appointed Data Safety and Monitoring Board. Causality was assessed by three independent reviewers using standardized DILIN likelihood scores (definite, highly likely, or probable). Discrepancies were resolved by consensus. This analysis included 2207 high-confidence DILI cases enrolled between 2003 and 2024.

### 2.1 Histology

Liver biopsies were reviewed by a single expert liver histopathologist (DEK) and scored for multiple histological features as well as an overall pattern as previously described^46^.

### 2.2 Statistical analysis

Descriptive statistics, including the median and interquartile range, were used to summarize continuous variables, while frequencies and percentages were used for categorical variables. Non-parametric tests, such as the Wilcoxon test for two groups and the Kruskal–Wallis test for three groups, were used to compare continuous variables. For categorical variables, Chi-square test or Fisher’s exact test for variables with low frequencies were used. All reported *P*-values are two-sided, with a significance level of ≤0.05 considered statistically significant. Data analysis was performed using SAS 9.4.

### 2.3 Genetic analysis

*HLA* data were available for 44 anti-HTN DILI cases and compared across ancestries to 1447 persons with non-anti-HTN DILI (as disease controls). *HLA* sequencing, performed on consented DILIN cases using the Illumina MiSeq platform at Vanderbilt’s Immunogenomics Core, followed methods described by Li et al^34^. SNP imputation was performed as previously described^47^. Population stratification of cases and controls was inferred by EIGENSTRAT as described^34^ (Supporting Information Fig. S1). SNP and *HLA* association analyses were performed by drug/class and ancestry using a fixed case-to-control ratio. Associations were assessed *via* allele frequency comparisons using logistic regression (adjusting for diabetes and the first two principal components) or Fisher’s exact test for groups with <30 cases. False discovery rate (FDR) was controlled using *q*-values, with *q* < 0.05 considered significant^47^^,^^48^. Rare *HLA* alleles, <0.05 allele frequency among cases, were excluded from the association analysis. Conditional analysis on the most significant *HLA* allele was performed. *HLA* haplotype analysis was performed by haplo.stats software^49^.

To verify the genetic associations, we used 5456 general population individuals obtained by merging five database of Genotypes and Phenotypes (dbGaP) datasets (see Supporting Information) using a fixed ratio of 124 between anti-HTN DILI cases and population controls and matched by discretized race and gender, and age and principal components (PC)1 to 4 within 2 SD (see below) using the *matchit R* function. For dbGaP controls in the current study, four-digit *HLA* alleles were imputed by the HLA Genotype Imputation with Attribute Bagging (HIBAG) program using available genotype data^50^. Association analyses were performed as reported above, with logistic regression adjusted only for the first two PCs.

## 3 Results

### 3.1 DILI from antihypertensives

Among 2207 cases of high-confidence DILI enrolled between 2003 and 2024, 44 (2%) were attributed to anti-HTN medications grouped into five distinct drug groups: hydralazine (*n* = 11), methyldopa (*n* = 13), ACE inhibitors and angiotensin receptor blockers (ACE/ARB) (*n* = 14), beta blockers (BB) (*n* = 3), and CCB (*n* = 3). The BB group consisted of 3 cases, one each attributed to atenolol, carvedilol, and labetalol. The ACE/ARB group included 14 cases, including 6 ascribed to lisinopril, 2 to benazepril/hydrochlorothiazide (HCTZ), 2 to losartan, and 1 each to irbesartan, and valsartan. All 3 cases ascribed to CCB were attributed to verapamil.

There was significant variability across the five groups. Patient demographics comparing the five groups are summarized in Table 1. Patients with methyldopa induced liver injury were on average younger [mean 31 years] compared to other groups [means ≥ 57 years]. Among the 13 patients with methyldopa-induced liver injury, 6 had gestational HTN and preeclampsia and 7 had pre-existing HTN and were treated with methyldopa during pregnancy. Gender distribution also showed notable differences, with the methyldopa and CCB groups consisting entirely of female patients (Table 1).

**Table 1:** Selected demographic, clinical, laboratory studies, and outcomes of 44 patients with DILI ascribed to anti-hypertensive medications.

Characteristic	Hydralazine (*n* = 11)	Methyldopa (*n* = 13)	Beta blocker (*n* = 3)	ACE/ARB (*n* = 14)	CCB (*n* = 3)
Age mean (SD)	61 (12.91)	31 (7.09)	59 (11.93)	57 (11.47)	64 (5.47)
Female sex	64%	100%	33%	36%	100%
Self-reported race					
White or Caucasian	64%	31%	67%	86%	100%
Non-hispanic black	36%	62%	0%	14%	0%
Hispanic or Latino	9%	8%	33%	7%	0%
Latency mean days (SD)	169 (262)	85 (53)	85 (90)	195 (226)	85 (83)
Recovery ≥6 months	30%	18%	0%	23%	0%
Signs and symptoms at DILI onset					
Jaundice	55%	77%	67%	71%	33%
Nausea	46%	62%	33%	43%	33%
Fever	9%	23%	33%	14%	0%
Abdominal pain	36%	46%	0%	29%	67%
Rash	0%	23%	33%	36%	0%
Pruritus	36%	54%	33%	71%	67%
Asymptomatic	9%	15%	0	0	0
Pattern of liver injury at DILI onset					
Cholestatic	27%	0%	0%	54%	33%
Hepatocellular	55%	100%	100%	31%	67%
Mixed	18%	0%	0%	15%	0%
Severity					
Mild	27%	15%	33.3%	21%	67%
Moderate	0	9%	0%	29%	33%
Moderate hospitalized	27%	61%	33.3%	28%	0%
Severe	37%	15%	33.3%	7%	0%
Liver transplant	9%	0%	0%	8%	0%
Death	0%	0%	0%	7%	0%

The latency period, defined as the time from drug initiation to the onset of DILI, showed considerable variation as well. Hydralazine DILI exhibited the longest median latency period (108 days, IQR 17–167), whereas CCB users had the shortest median latency (44 days, IQR 30–180). The pattern of liver injury also differed across the groups. Hepatocellular injury was universally observed in the methyldopa and beta blocker groups. In contrast, cholestatic injury was prevalent among ACE/ARB users (53.8%), with only a third presenting as hepatocellular and 15% as mixed (Table 1). The hydralazine and ACE/ARB groups showed the slowest recovery, with 30% and 23% of patients, respectively, still exhibiting abnormal liver tests at 6 months. Severity of DILI varied among the groups and was most severe in the hydralazine, methyldopa, BB, and ACE/ARB cases. Two patient required a liver transplant, one with hydralazine DILI and one who received lisinopril (Table 1). One patient with lisinopril DILI died (Supporting Information Tables S2–S5).

### 3.2 Histological findings

Liver tissue from 16 subjects were available for review, including 15 needle biopsies and 1 explant (Table 2 and Fig. 1. The specimens were obtained a median of 13 days after onset (range 2 to 84 days). A variety of histological injury patterns were observed. There were three biopsies of DILI due to methyldopa, all with slightly different injury patterns (Table 2). Two showed severe portal and pan-lobular hepatitis, one displayed moderate canalicular cholestasis. The third case showed cholestatic injury with only minimal inflammation despite HC-type injury from lab results. Lisinopril was the only ACE inhibitor among the biopsied cases, which showed cholestatic injury, with varied degrees of portal and lobular lympho-histiocytic inflammation. In addition, the explanted liver from the patient with lisinopril DILI who underwent transplantation revealed underlying cirrhosis from steatohepatitis along with cholestasis (the patient had metabolic risk factors). There were 5 cases of hydralazine DILI, all of which showed moderate to severe hepatitis with necrosis that varied from mild to complete hepatic necrosis.

**Table 2:** Histological findings in the 16 patients with available liver biopsies.

Drug	Days after onset	Ishak scores grade/stage	Plasma cell/eosinophils	Necrosis	Bile stasis	Other finding	Descriptive diagnosis
Atenolol	3	10/0	−/+	None	None	Granuloma	Granulomatous hepatitis
Labetolol	84	15/2	−/−	Mild	None		Acute hepatitis
Methyldopa	5	9/2	−/+	None	None		Acute hepatitis
Methyldopa	14	16/0	+/−	Moderate	++		Acute cholestatic hepatitis
Methyldopa	2	11/3	−/−	Minimal	+	Chronic cholestatic features, early bridging fibrosis *vs* reticulin collapse	Acute hepatitis
Enalapril	76	4/2	−/−	None	+++	Chronic cholestasis with possible duct loss, extensive perisinusoidal fibrosis	Acute and chronic cholestatic injury
Irbesartan	10	5/0	−/−	Minimal	None	Sinusoidal lymphocytosis	Chronic (portal) hepatitis
Lisinopril	24	6/0	−/−	Minimal	+++	Moderate steatosis	Acute cholestatic injury
Lisinopril	42	9/1	−/+	None	+		Cholestatic hepatitis
Lisinopril-hydrochloro-thiazide	25^b^	8/6	−/−	None	+++	Steatohepatitis	Acute cholestasis, superimposed on cirrhosis steatohepatitis
Losartan	11	5/2	−/+	None	++		Chronic cholestatic injury
Hydralazine	73	17/3	+/+	Mild	None	Hepatocyte giant cells	Acute hepatitis
Hydralazine	22	13/0	−/+	Mild	None		Hepatitis with zone 3 necrosis
Hydralazine	6	a/a	+/−	Severe			Massive necrosis
Hydralazine	3	17/2	+/−	Moderate	None		Hepatitis with zone 3 necrosis
Hydralazine	8	12/0	−/+	Moderate	+		Hepatitis with zone 3 necrosis

a Could not be assessed due to complete hepatocellular necrosis.

b Explanted liver.

**Figure 1:**
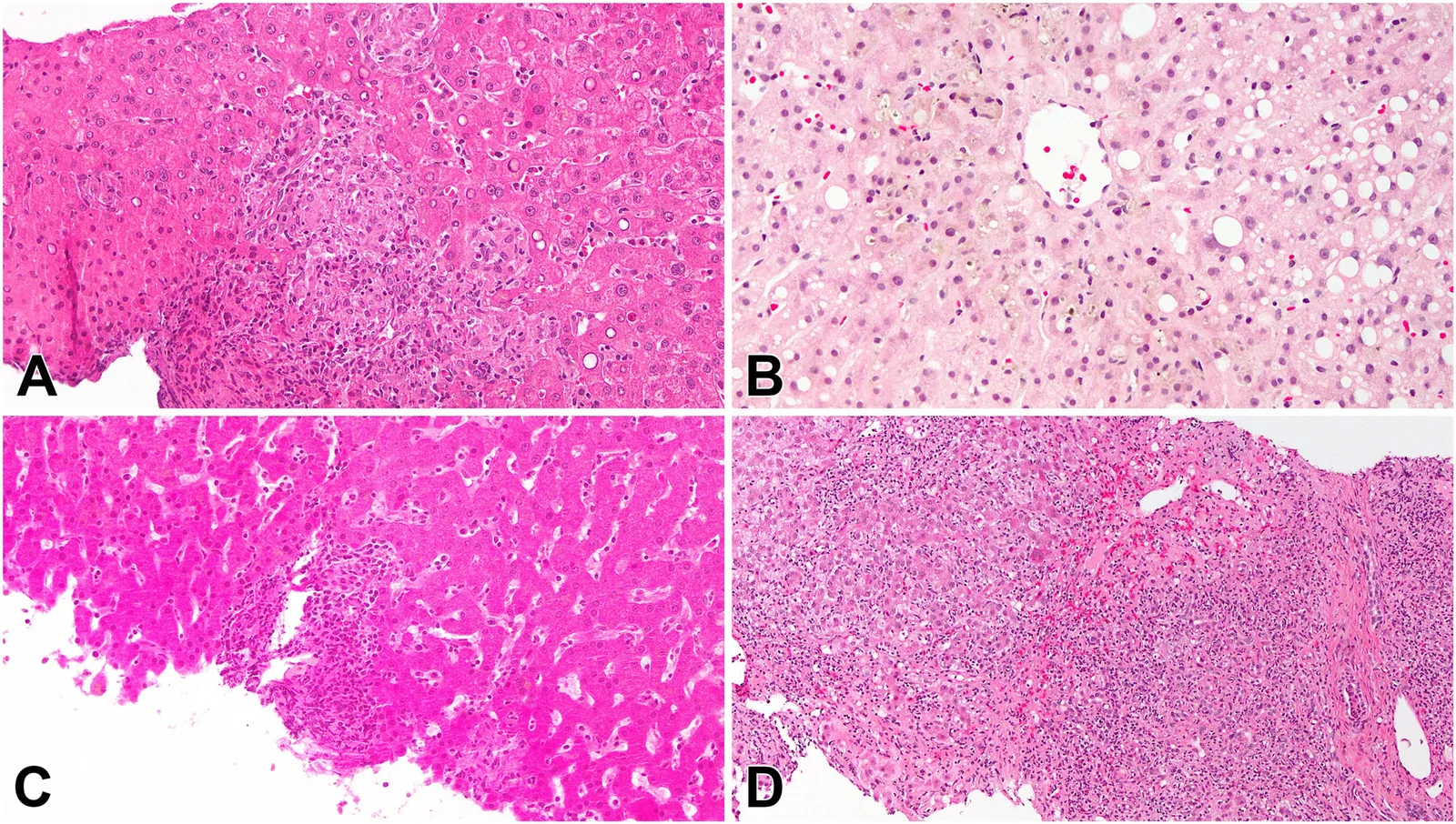
Examples of histological injury from anti-hypertensive medications. (A) Granulomatous hepatitis associated with atenolol. There are multiple irregular granulomas involving portal areas and parenchyma (H&E, 200×). (B) Lisinopril-induced cholestasis. There is mild steatosis, and both hepatocellular and canalicular cholestasis in zone 3 (H&E, 200×). (C) Hepatitis associated with irbesartan. There is mild chronic portal inflammation with disruption of the limiting plate. The adjacent sinusoids show an infiltrate of lymphocytes and macrophages (H&E, 200×). (D) Hepatitis with areas of confluent necrosis associated with hydralazine. There is marked portal and parenchymal inflammation with an area of hepatocyte loss and marked ductular reaction (H&E, 100×).

### 3.3 Comparison of hydralazine and methyldopa

Comparison between the two single agents most frequently implicated, hydralazine and methyldopa, revealed notable statistically significant demographic and clinical differences (Table 3). Among the patients with methyldopa DIILI Antinuclear Antibodies (ANA) was positive in 53% (7/13); Anti-Smooth Muscle Antibodies (ASMA) positive in 39% (5/13). Among those with hydralazine DILI ANA was positive in 46% (5/11) and ASMA in 18% (2/11). Of the 13 methyldopa patients, all were young women, 4 were pregnant, 5 were post-partum, and 4 women were neither. Patients in the hydralazine group were significantly older (*P* < 0.001). Unsurprisingly, because methyldopa is now prescribed chiefly to pregnant or post-partum women, the methyldopa group was 100% female compared to hydralazine (63%) (*P* = 0.031). The pattern of liver injury was 100% hepatocellular in methyldopa *vs.* 54% in the hydralazine group (*P* = 0.011). Among the cases reported to DILIN, both hydralazine (64%) and methyldopa (77%) caused rather severe injury requiring hospitalization but only hydralazine resulted in liver transplant (Table 3 and Supporting Information Table S3).

**Table 3:** Selected features of DILI ascribed to hydralazine *vs* methyldopa.

Characteristic	Hydralazine *n* = 11	Methyldopa *n* = 13	*P*-value
Age mean (SD)	60.5 (12.91)	31.4 (7.09)	<0.001
Female sex	64%	100%	0.031
Caucasian	66%	31%	
Black	36%	62%	
Asian	0%	0%	
Other/Multiracial	0%	8%	
Latino	9%	8%	>0.999
Latency mean (SD)	168.6 (262)	84.5 (53)	0.417
Labs at DILI onset			
ANA+	45.5%	54.5%	0.670
ASMA+	18.2%	36.4%	0.635
ALT (U/L) mean (SD)	958 (936)	1467 (751)	0.060
AST (U/L) mean (SD)	936 (641)	1486 (641)	0.046
Alkaline phosphatase (U/L) mean (SD)	283 (147)	144 (70)	0.003
Total bilirubin (mg/dL) mean (SD)	7.9 (9.3)	9.2 (6.1)	0.284
Pattern of liver injury at DILI onset			
Cholestatic	27.3%	0%	0.011
Mixed	18.2%	0%	
Hepatocellular	54.5%	100.0%	
Recovery from peak to below ULN (days)	70.0	39.0	0.344
Outcome			
All death	9.1%	0%	0.478
Liver transplant	9.1%	0%	0.458
Persistent injury >6 months	30.0%	18.2%	0.635
Severity score			
Mild-moderate	27%	23%	0.270
Hospitalized/Severe	64%	77%	
Fatal (LT or death)	9%	0%	

All continuous variables expressed as median (Q1, Q3) unless stated otherwise.

### 3.4 Relative risks of DILI due to anti-hypertensive medications

By dividing the number of high-confidence cases of DILI ascribed to anti-HTN medications by the number of persons prescribed such drugs in the USA, it is possible to estimate the relative risks of DILI and to compare the relative risks of the several drugs. We have performed such analyses recently for other drug classes^51^^,^^52^. Supporting Information Table S6 summarizes results for anti-HTN drugs. Most striking is the very high relative risk of DILI due to methyldopa (>81.8 cases/1 million persons prescribed). The second highest relative risk is that of hydralazine, albeit more than an order of magnitude less than that of methyldopa (6.8/1 million). Also notable are several frequently prescribed drugs that have no cases of DILI enrolled in the US DILIN over the past 20 years, including HCTZ, metoprolol, amlodipine, furosemide, diltiazem, propranolol, and spironolactone, as recently pointed out also by Chen et al^52^. Thus, many anti-HTN drugs are remarkably safe for the liver and may be preferable to methyldopa or hydralazine, verapamil, and enalapril.

### 3.5 Genetic analysis

Forty four high confidence anti-HTN DILI cases (24 Non-Hispanic White (NHW), 16 Non-Hispanic Black (NHB), and 4 Hispanic (HISP)) were compared to 1447 persons with DILI ascribed to drugs other than anti-HTN (1058 NHW, 181 NHB, 134 HISP). Significant *HLA* association results are reported in Table 3. In the genome-wide analysis of the multi-ethnic cohort, *HLA-B∗35:01* was significantly associated with an increased risk of liver injury due to any anti-HTN medications. Half of the anti-HTN DILI cases carried *HLA-B∗35:01* compared to only 11% of controls (Table 4). Across the entire cohort (*n* = 44), HLA-B∗35:01 had a sensitivity of 50% and a specificity of 99% relative to population controls, with a Positive Predictive Value (PPV) of 0.02 and an Negative Predictive Value (NPV) of 0.87.

**Table 4:** Summary statistics of the most significant HLA alleles comparing antihypertensive-DILI cases to DILI ascribed to non-antihypertensive drugs (non-anti-HTN DILI) across ethnicities#.

*HLA* allele	Anti-HTN CF	Non-anti-HTN CF	OR (95% CI)	*P*
Overall (Anti-HTN *n* = 44; non-anti-HTN DILI *n* = 1447)				
*B*^b^*35:01*	0.50	0.11	6.0 (3.4-10.6)	1 × 10-8^b^
*C*^b^*04:01*	0.59	0.24	2.5 (1.5-4.0)	0.0004^b^
*DQA1*^b^*01:01*	0.48	0.26	2.3 (1.3-3.8)	0.003^b^
*DQB1*^b^*05:01*	0.43	0.23	2.0 (1.2-3.4)	0.01^c^
ACEI + ARB (*n* = 14*;* non-anti-HTN DILI *n* = 1447)^a^				
*B*^b^*35:01*	0.43	0.11	5.0 (1.5-12.1)	0.004
*C*^b^*04:01*	0.43	0.24	2.2 (0.8-5.5)	0.08
*DQA1*^b^*01:01*	0.36	0.26	1.7 (0.6-4.3)	0.27
*DQB1*^b^*05:01*	0.29	0.23	1.6 (0.5-4.3)	0.38
Methyldopa (*n* = 13*;* non-anti-HTN DILI *n* = 1447)^a^				
*B*^b^*35:01*	0.69	0.11	9.0 (3.5-21.8)	8 × 10-6^b^
*C*^b^*04:01*	0.85	0.24	5.7 (2.4-13.5)	4 × 10-5^b^
*DQA1*^b^*01:01*	0.62	0.26	2.7 (1.0-6.6)	0.02
*DQB1*^b^*05:01*	0.54	0.23	2.7 (0.9-6.7)	0.03
Hydralazine (*n* = 11*;* non-anti-HTN DILI *n* = 1447)^a^				
*B*^b^*35:01*	0.27	0.11	2.7 (0.5-9.3)	0.12
*C*^b^*04:01*	0.36	0.24	1.5 (0.4-4.5)	0.52
*DQA1*^b^*01:01*	0.27	0.26	1.0 (0.2-3.3)	>0.99
*DQB1*^b^*05:01*	0.27	0.23	1.2 (0.2-3.9)	0.74

#Results were from the logistic regression model matched by genetic ancestry, gender, age within 10 years, and PC1 to PC2 within 2 standard deviations, adjusted for PC1 and PC2, and adjusted for diabetes status for Non-Anti-HTN DILI cases.

a Results were from Fisher’s exact test. Case:Control Ratio 1:12 for population controls, 1:5 for Non-Anti-HTN DILI subjects.

b *Q* < 0.05.

c *Q* < 0.15 Abbreviations: OR-odds ratio; CI- confidence interval.

*HLA-B∗35:01* was associated with a 6-fold increased risk of DILI (OR = 6.0 [3.4–10.6] *P* = 1.0 × 10^−8^; *Q* = 3.9 × 10^−7^). Stratified analyses showed consistent effect sizes across racial groups, with carrier frequencies of 50% *vs.* 11% in NHW, 43% *vs.* 9% in NHB, and 69% *vs.* 13% in Hispanic individuals (Supporting Information Table S8). Control frequencies were similar across groups, indicating no single ethnicity drove the association *HLA-B∗35:01, HLA-C∗04:01, HLA-DQA1∗01:01,* and *HLA-DQB1∗05:01* were also significantly associated with an increased risk of DILI from anti-HTN medications exceeding the multiple correction threshold (Table 4). A conditional analysis was carried out to assess the possible effect size of each allele independent from *HLA-B∗35:01* (Supporting Information Table S7). The analysis showed none of the most significant *HLA* alleles was independent from *HLA-B∗35:01.* The effect sizes of *HLA-C∗04:01, HLA-DQA1∗01:01,* and *HLA-DQB1∗05:01* were consistent among ethnicities with similar carrier rates (Table S8). Haplotype analysis showed that all possible haplotypes imputed in the cohort, including *HLA-B∗35:01* and at least another allele among the significant ones, were strongly significant within the multiethnic population control population (Table S8). Being a carrier for the four risk alleles (13% in cases and 2% in controls) was very strongly associated with the risk of developing DILI (*P* = 4 × 10^−13^). Carriage of *HLA-B∗35:01* was not associated with any specific phenotype or severity of DILI, but importantly was associated with a shorter latency (50 *vs*. 109 days, *P* = 0.02) (Table 5).

**Table 5:** Selected demographic, clinical, laboratory studies, and outcomes of 44 patients with DILI ascribed to anti-hypertensive medications—Comparison of subjects without *vs.* with *HLA-B∗35:01* Carriage.

Clinical variable		*HLA-B∗35:01* Non-carrier (*n* = 22)	*HLA-B∗35:01* Carrier (*n* = 22)	*P*-value
AGE, mean (SD)		53.5 (14.8)	48.3 (17.7)	0.3
Female sex		55%	77%	0.2
Race				
Non-hispanic black		36%	36%	>0.99
Hispanic		9%	9%	
Non-hispanic white		55%	55%	
BMI, mean (SD)		31.1 (7.3)	32.2 (8.3)	0.8
LATENCY, mean (SD)		206.1 (240.1)	69.5 (53.1)	0.02
JAUNDICE		55%	77%	0.2
PHENOTYPE	Cholestatic	32%	23%	0.79
	Hepatocellular	59%	68%	
	Mixed	9%	9%	
PEAK ALT, mean (SD)		963.9 (998.2)	1209.1 (918.1)	0.23
PEAK AST, mean (SD)		885.3 (899.7)	1208.0 (807.3)	0.14
PEAK ALP, mean (SD)		514.7 (643.3)	374.9 (460.4)	0.37
PEAK TBILI, mean (SD)		11.3 (11.7)	15.7 (11.7)	0.23
PEAK INR, mean (SD)		1.5 (0.7)	1.4 (0.4)	0.48
TRANSPLANT		1 (5%)	1 (5%)	>0.99
SEVERITY				
Mild-moderate		46%	32%	0.27
Hospitalized/severe		50%	64%	
Fatal		5%	5%	

Non-anti-HTN DILI subjects showed a comparable carriage frequency to the general population for *HLA-B∗35:01* (11% in DILIN *vs.* 13% in the dbGaP population) and *HLA-C∗04:01* (24% *vs.* 27%), but a higher carriage frequency for *HLA-DQA1∗01:01* (26% *vs.* 18%). This finding confirmed that the top alleles were not associated with DILI as a phenotype, even though *HLA-DQA1∗01:01* could potentially be a shared risk factor across multiple causal drugs. Association analysis comparing anti-HTN DILI cases with population controls confirmed the findings (Supporting Information Table S9).

When stratified by major causal drug/class groups [hydralazine (11), methyldopa (13), and ACE-IRB (14)], *HLA-B∗35:01* carriage frequency was consistently higher among cases due to each of the anti-HTN classes/drugs (Table 4), confirming the shared risk association. A list of all the carriers of *HLA-B∗35:01* and their respective causal drugs is in Supporting Table S10. Methyldopa-DILI was the largest drug-specific group (13 individuals, 4 of whom were NHW, 8 NHB, and 1 Hispanic). *HLA-B∗35-01* was the strongest risk allele with 100% carriage frequency in NHW and Hispanic cases and 50% in NHB, and a more than nine-fold overall increase in DILI risk across all ethnicities (Table 4 and Supporting Information Table S11). *HLA-A∗68:02 and HLA-DRB1∗15:03* showed an effect independent from that of *HLA-B∗35:01* after conditional analysis, passing multiple testing corrections. Interestingly, *HLA-B∗53:01* was also found to be marginally significant (Supporting Information Table S12). No additional drug/class-specific *HLA* risk alleles passed the multiple testing correction.

Finally, we checked *HLA-DRB1∗04:01* and *HLA-DRB1∗03:01,* known risk alleles for autoimmune hepatitis^53^. Despite the fact that a significant difference in positive ANA or SMA frequencies was found between *HLA-B∗35:01* carriers and non-carriers, this family of alleles was not associated with DILI in our cohort or within drug subgroups (Supporting Information Tables S13 and S14).

## 4 Discussion

This paper describes and analyzes high-confidence cases of idiosyncratic DILI ascribed to antihypertensive medications enrolled in the US DILIN between September 2003 and September 2023. Methyldopa DILI occurred exclusively in young women (9 out of 13 pregnant or post-partum) and presented with hepatocellular injury. This reflects prescription practices where methyl dopa is mostly prescribed mainly for hypertension during pregnancy and in the post partum period^17^^,^^18^. Among other anti-HTN agents, 60% of cases were hepatocellular, 28% cholestatic, and 9% mixed—patterns consistent with the broader DILIN cohort^30^. Although rare, anti-HTN DILI was often severe: nearly half the patients developed serious liver injury, including two requiring liver transplantation and one fatality. Methyldopa accounted for 75% of hospitalizations and the highest ALT/AST elevations, likely reflecting its use in pregnancy, where hypertensive complications often lead to admission^23^. In contrast, verapamil DILI was uniformly mild and self-limited.

Genetic analysis identified *HLA-B∗35:01* in 50% of anti-HTN DILI cases, compared to 11% in non-anti-HTN DILI and 13% in population controls, conferring a fourfold increased risk and significantly shorter latency (50 *vs.* 109 days). This association was robust across multiple anti-HTN agents and ethnicities. Interestingly, *HLA-B∗35:01* has been associated with autoimmune hepatitis AIH in the Chinese population^54^, other autoimmune diseases^55^, and other drug-induced adverse events^56^. Moreover, an association with *HLA-B∗35:01* and its haplotype has been previously demonstrated for DILI caused by other drugs or herbal substances, such as *Polygonum multiflorum*^57^, green tea^29^, *Garcinia cambogia*^31^*,* turmeric^53^, Kampo medicines^54^, and trimethoprim/sulfamethoxazole^34^. Additional *HLA* alleles*, HLA-C0∗4:01, HLA-DQA1∗01:01, and DQB1∗05:01*, were also enriched, likely reflecting linkage disequilibrium within a previously reported haplotype^29^.

Within the cohort, *HLA-B∗35:01* had a sensitivity of 50% and specificity of 99% (PPV: 0.02; NPV: 0.87); sensitivity rose to 69% in methyldopa DILI. *HLA-B∗53:01*, a related MHC molecule, showed a nominal association with methyldopa DILI independent of B∗*35:01*, particularly among non-Hispanic Black patients, along with a rare case of *HLA-B∗*35:08 (Supporting Information Table S15). Functionally, *HLA-B∗35:01* is characterized by increased peptide receptivity at the cell surface and within acidified vesicles^37^ and an enhanced capacity for cross-presentation of endogenous and exogenous antigens, which may underlie its recurrent association with DILI from diverse agents. Notably, none of the patients in this cohort reported use of herbal products, and the potential interaction between such agents and anti-hypertensive medications remains unknown.

Among all anti-HTN drugs*, HLA-B∗35:01* was the strongest predictor for methyldopa DILI, associated with a seven-fold increased risk. As methyldopa is primarily used during pregnancy and is known to trigger autoimmune-like DILI, the predominance of pregnant women (69%) in this subgroup is unsurprising^23^. Importantly, *HLA-B∗35:01*carriage did not differ between pregnant and non-pregnant women, reinforcing the drug-specific association. Methyldopa remains on the WHO list of essential medicines, as it is commonly used for hypertension during pregnancy worldwide^19^. Our findings suggest its use should be restricted due to this risk and when its use in necessary, pre-prescription HLA testing may be clinically valuable given the high specificity and negative predictive value of *HLA-B∗35:01*. The specificity of this HLA testing may be diminished in patients exposed to multiple agents that share this genetic risk (such as trimethoprim/sulfamethoxazole), underscoring the need to interpret HLA associations within the broader clinical, exposure, and temporal context.

An important limitation of our study is the small sample size, reflecting the rarity of antihypertensive-associated DILI. In addition, we acknowledge that we cannot entirely rule out an association between *HLA-B∗35* itself and hypertension or a disease and drug interaction associated with DILI in the presence of *HLA-B∗35:01*. Studies on animal models^58^^,^^59^ have hypothesized that hypertension might have an autoimmune component since antigenic isolevuglandin-adduced peptides restricted by specific *HLA* class I molecules have a role in the development of hypertension. However, large genetic studies in patients with systemic arterial hypertension have not revealed any relevant association of *HLA* alleles or variants in the MHC regions^60^. In addition, a genome-wide association study examining *HLA* allele associations across multiple complex diseases did not show any significant signals between specific class I *HLA* alleles and hypertension^61^. Futhermore, *HLA-B∗35:01* has been previously associated with DILI due to other prescription drugs and herbal products used for other indications, supporting a strong association with hepatotoxicity and DILI, rather than a result of confounding by indication bias^33^^,^^34^^,^^39^^,^^40^.

## 5 Conclusions

In conclusion, HLA-B∗35:01 may serve as a predictive marker for anti-HTN DILI, particularly methyldopa-induced liver injury. Incorporating pharmacogenetic screening into clinical practice could help identify at-risk individuals and mitigate adverse outcomes. Importantly, our findings also provide a foundation for mechanistic studies exploring how this allele confers susceptibility to liver injury across structurally diverse agents.

## Author contributions

Lily Dara: conceptualization, writing original draft, editing, and funding acquisition. Herbert L. Bonkovsky: conceptualization, editing and funding acquisition. Paola Nicoletti: methodology, formal analysis and writing original draft. Andrew Dellinger: methodology, formal analysis writing review and editing, data curation. Mahmoud G. Mohamed: writing review and editing, data curation. Chaudry Nasir Majeed: writing review and editing, data curation. David E. Kleiner: data curation, writing review and editing. Tatyana Kushner: writing review and editing. Raj Vuppulanchi: writing review and editing. Elizabeth Phillips: writing review and editing. David A. Ostrov: writing review and editing. Mohammed Umair Masood: writing review and editing. Huiman Barnhart: methodology, formal analysis, data curation, funding acquisition. Jiezhun Gu: data curation and formal analysis.

## Conflicts of interest

The authors declare no relevant financial conflict of interest regarding this manuscript.

## Appendix A Supporting information

The following is the Supporting Information to this article:
