# Supplementary material for: Antihypertensive drug-induced liver injury is rare, but strongly associated with HLA-B∗35:01
Source: Acta Pharm Sin B. 2026 Jun 10;16(10):6676–85. doi: 10.1016/j.apsb.2026.06.003 (PMC13712353; doi:10.1016/j.apsb.2026.06.003)
Supplement: Supplementary file 1 [file mmc1.docx]

**Supporting Information for**

**Original article**

**Antihypertensive drug-induced liver injury is rare, but strongly associated with HLA-B*35:01**

**Lily Dara^a,*^, Paola Nicoletti^b^, Chaudry Nasir Majeed^j^, Andrew Dellinger^h^, David E. Kleiner^i^, Mahmoud G. Mohamed^a^, Tatyana Kushner^c^, Raj Vuppulanchi^f^, Elizabeth Phillips^d^, David A. Ostrov^e^, Mohammed Umair Masood^b^, Huiman Barnhart^g^, Jiezhun Gu^g^, Herbert L. Bonkovsky^j^**

^a^*Division of Gastrointestinal and Liver Diseases, Research Center for Liver Diseases, Department of Medicine, Keck School of Medicine of the University of Southern California, Los Angeles, CA 90033, USA*

^b^*Department of Genetics and Genomic Sciences, Icahn School of Medicine at Mount Sinai, New York, NY 10029, USA*

^c^ *Department of Gastroenterology and Hepatology, Weill Cornell Medicine, New York, NY 10065, USA*

^d^*Department of Medicine, Vanderbilt University Medical Center, Nashville, TN 37232, USA*

^e^*Department of Pathology, Immunology and Laboratory Medicine, University of Florida College of Medicine, Gainesville, FL 32610, USA*

^f^*Division of Gastroenterology and Hepatology, Department of Medicine, Indiana University School of Medicine and Indiana University Health, Indianapolis, IN 46202, USA*

^g^*Duke Clinical Research Institute, Durham, NC 27710, USA*

^h^*Duke Molecular Physiology Institute, Duke University School of Medicine, Durham, NC 27710, USA*

^i^*Laboratory of Pathology, National Cancer Institute, Bethesda, MD 20892, USA*

^j^*Section on Gastroenterology and Hepatology, Department of Medicine, Wake Forest University School of Medicine and Advocate Health Wake Forest Baptist, Winston-Salem, NC 27101, USA*

Receive 8 December 2025; received in revised form 30 January 2026; accepted 9 February 2026

^*^Corresponding author.

E-mail address: [lily.dara@usc.edu](mailto:lily.dara@usc.edu) (Lily Dara).

1. **Supporting methods**

**Control cohorts for genetic analysis**: To assemble the population control dataset, we obtained approval from NCBI dbGaP to access the following datasets: 1) eMERGE-I: Genome Wide Association Studies of Network Phenotypes (phs000360.v3.p1); 2) KORA: Genetic Epidemiology of Refractive Error in the KORA study (phs000303.v1.pl); 3) KIDRISK: Pooled Genome-Wide Analysis of Kidney Cancer Risk (KIDRISK, phs001271.v1.p1); 4) Ashkenazi: Genetics of Schizophrenia in an Ashkenazi Jewish Case-Control Cohort (phs000448.v1.p1); and 5) BioMe: The Charles Bronfman Institute for Personalized Medicine BioMe BioBank (phs000925.v1.p1). The population control dataset includes all subjects in eMERGE-1 and BioMe, controls from KIDRISK and Ashkenazi, and subjects without high myopia (defined by refractive error < -5 diopter of at least one eye) from KORA. eMERGE1, KORA, KIDRISK, and Ashkenazi control sets were used to match the NHW AH-DILI cases and BioMe was used to match both NHB and HSP AH-DILI cases.

**Specific acknowledgments:** We thank NCBI/dbGaP at <http://www.ncbi.nlm.nih.gov/gap>, who approved our application to obtain additional control datasets. In particular, eMERGE GWAS datasets were from dbGaP accession number phs000360.v3.p1. The eMERGE network is a consortium of five participating sites (Group Health Seattle, Marshfield Clinic, Mayo Clinic, Northwestern University, and Vanderbilt University) funded by the National Human Genome Research Institute (NHGRI). Funding supports for the cohort and genotyping of eMERGE samples are as follows: (1) Group Health Seattle: U01AG006781 and U01HG004438 with genotyping performed at Johns Hopkins University; (2) Mayo Clinic: UOIHG004599 and U01HG004424 with genotyping performed at The Broad Institute; (3) Marshfield Clinic: U01HG004608 and U01HG004438 with genotyping performed at Johns Hopkins University; (4) Northwestern University: U01HG04603 and U01HG004424 with the genotyping performed at The Broad Institute; (5) Vanderbilt University: U01HG004603 and U01HG004424 with the genotyping performed at The Broad Institute. The PAGE dataset is from the Charles Bronfman Institute for Personalized Medicine (IPM); the BioMe BioBank is from the Icahn School of Medicine at Mount Sinai (New York, NY). Phenotype data collection was supported by The Andrea and Charles Bronfman Philanthropies. Funding support for genotyping, which was performed at The Center for Inherited Disease Research (CIDR), was provided by the NIH (U01HG007417). The GWAS data were from dbGaP accession number phs000925.v1.p1. The Ashkenazi GWAS data were from dbGaP accession number phs000448.v1.p1. The dataset was provided by Dr. Todd Lencz and Ariel Darvasi, Ph.D. Support for collection and analysis was provided by RC2MH089964, R01 MH084098, the North Shore - LIJ Health System Foundation, and the Hebrew University Genetic Resource. The kidney GWAS data were from dbGaP accession number phs001271.v1.p1. The dataset was funded by grant NCI U01CA155309 to G Scelo. The KORA GWAS data were from dbGaP accession number phs000303.v1.pl. The dataset was obtained from the National Eye Institute Refractive Error Collaboration (NEIREC) Database. Funding support for NEIREC was provided by the National Eye Institute. We thank NEIREC participants and the NEIREC Research Group for their valuable contribution to this research.

2. **Supporting figures**
a. All Races

**Figure S1** Cases and Controls distribution for multiethnic Anti-HTN DILI cohort plotting PC1 and PC2

3. **Supporting tables**

**Table S1** Summary descriptions of previously reported cases of idiosyncratic DILI ascribed to anti-hypertensive medications [Limited to reports written in English]

| **Calcium channel blockers** | | |
| --- | --- | --- |
| 2022 (Yet Kwong Horman, Patel et al. 2022) | Drug | Amlodipine |
|  | Demographics | 88 y/o F |
|  | Phenotype | Hepatocellular, ALF |
|  | Latency | 21 D |
|  | Liver enzymes | ALP 234 U/L, AST 1032 U/L, ALT 1247 U/L, TB 2.5 mg/dL, R= 13 |
|  | Histology | NR |
|  | Outcome | Back to normal by 53 D |
| 2020 (Varghese, Madi et al. 2020) | Drug | Amlodipine, 5 mg po daily |
|  | Demographics | 47 y/o M |
|  | Phenotype | Hepatocellular |
|  | Latency | 4 D |
|  | Liver enzymes | ALT 449 U/L, AST 271 U/L, TB 0.87 mg/dL, R= NR |
|  | Histology | NR |
|  | Outcome | Improved 5 days post-discontinuation |
| 2015 (Hammerstrom 2015) | Drug | Amlodipine, 5 mg PO daily |
|  | Demographics | 34 y/o Caucasian M |
|  | Phenotype | Hepatocellular |
|  | Latency | 2 D |
|  | Liver enzymes | AST 269 IU/L, ALT 630 IU/L, ALP & TB ‘within normal limits’, NR. R=15.8 |
|  | Histology | Hepatocellular injury with a possibility of drug injury or another toxin |
|  | Outcome | Improved within a week of stopping |
| 2013 (Demirci, Polat et al. 2013) | Drug | Amlodipine, 10 mg PO daily |
|  | Demographics | 46 y/o M |
|  | Phenotype | Hepatocellular |
|  | Latency | Within 7 D |
|  | Liver enzymes | TB 1.44 mg/dL, AST 519 U/L, ALT 923 U/L, ALP U/L, 102 (38-155) U/L, GGT 521 U/L, R = 27.2 |
|  | Histology | Lesions consistent with drug-induced hepatotoxicity |
|  | Outcome | Normalized within 3 weeks post-discontinuation |
| 2009 (Chaudhry, Maqsood et al. 2009) | Drug | Nicardipine, dose NR |
|  | Demographics | 61 y/o M |
|  | Phenotype | Mixed |
|  | Latency | 4 D |
|  | Liver enzymes | AST 450 U/L, ALT 356 U/L, ALP 299 U/L, serum TB 0.6 mg/dL. R = 3.6 |
|  | Histology | NR |
|  | Outcome | Improved few days after stopping Nicardipine. |
| **Beta blockers** | | |
| 2020 (Whelan, Izewski et al. 2020) | Drug | Labetalol, dose NR |
|  | Demographics | 39 y/o pregnant F |
|  | Phenotype | Hepatocellular |
|  | Latency | 210 D |
|  | Liver enzymes | ALT 206 U/L, AST 524 U/L, ALP 86 U/L, T Bili 0.5 mg/dl, R = 7.1 |
|  | Histology | Normal liver architecture without fibrosis, steatosis, or cholestasis. No viral inclusions, inflammatory cells were predominantly mononuclear without large numbers of eosinophils. No autoimmune hepatitis, HELLP or AFLP. No centrilobular necrosis was observed. |
|  | Outcome | Improved after discontinuation and almost back to normal by term. Despite insignificant pathology findings, considering prompt improvement in liver enzymes after discontinuation supported the diagnosis of drug-induced liver injury. |
| 2019 (Mitchell, Harner et al. 2019) | Drug | Carvedilol 12.5 mg/day; Metoprolol succinate 25 mg/day |
|  | Demographics | 30 y/o White M |
|  | Phenotype | Hepatocellular |
|  | Latency | 30 D after carvedilol .2 D of Metoprolol. |
|  | Liver enzymes | Carvedilol: AST 2686 U/L, ALT 2494 U/L, INR 2.5, TB 6.9 mg/dl, R = NR  Metoprolol: AST 1326 U/L, ALT 968 U/L, TB 6.6 mg/dL, R = NR |
|  | Histology | N\R |
|  | Outcome | Improved after 2–3 days of discontinuation. |
| 2018 (Rua, Prata et al. 2019) | Drug | Carvedilol 12.5 mg/day. |
|  | Demographics | 83 y/o F |
|  | Phenotype | Cholestatic then mixed |
|  | Latency | NR |
|  | Liver enzymes | AST, ALT, ALP, GGT 67 U/L,110 U/L, 430 U/L, 936 U/L, R = 0.77 at presentation and 453 U/L, 1,098 U/L, 230 U/L, and 316 U/L, R =14.3 at 5^th^ to 13^th^ day of hospitalization. |
|  | Histology | Mixed pattern of liver injury with hepatocellular inflammation and necrosis and mild signs of chronic cholestasis |
|  | Outcome | Improved after a month of discontinuation and back to normal in 9 months |
| 2017 (Philips, Paramaguru et al. 2017) | Drug | Metoprolol,100 mg/day |
|  | Demographics | 56 y/o F |
|  | Phenotype | Hepatocellular |
|  | Latency | 60 D |
|  | Liver enzymes | Peaked at AST 1823 U/L, ALT 2262 U/L, TB 24.8 mg/dlL ALP 224 IU/L, INR 3.82, Ammonia 238 μmol/L R = 30.3 |
|  | Histology | Extensive bridging necrosis with islands of normal surrounding hepatocytes and evidence of mixed inflammatory infiltrate (neutrophils and lymphocytes and occasional plasma cells) with early fibrous laying down more within areas of necrosis suggestive of acute hepatitis with bridging necrosis secondary to drug insult. |
|  | Outcome | Started to improve after a week and close to normal at 3–4 weeks post-discontinuation and plasmapheresis |
| 2015 (Firoz, Webber et al. 2015) | Drug | Labetalol, Methyldopa, dose NR |
|  | Demographics | 39 y/o 20 wks pregnant F |
|  | Phenotype | Hepatocellular |
|  | Latency | Methyldopa: 56 D, Labetalol: 28 D |
|  | Liver enzymes | ALT 1406 U/L, AST 2701 U/L, ALP 159 U/L, GGT 37 U/L, TB 16.6 mg/dL, INR 3.1, R = 26.5 |
|  | Histology | Acute liver injury consistent with drug-/toxin-induced liver injury. |
|  | Outcome | Started to improve after a week of cessation |
| 2009 (Dumortier, Guillaud et al. 2009) | Drug | Atenolol, 100 mg/day |
|  | Demographics | 57 y/o F |
|  | Phenotype | Hepatocellular |
|  | Latency | 30 D post liver transplant |
|  | Liver enzymes | AST 351 U/L, ALT 789 U/L, ALP 278 U/L, GGT 244 U/L, TB 2.3 mg/dL, INR 1.0, R = 8.51 |
|  | Histology | Large areas of confluent necrosis were observed in centrilobular areas; they were surrounded by inflammatory cells, including lymphocytes and plasma cells, pigmented macrophages, and scattered hepatocellular apoptotic bodies suspected toxic hepatitis. |
|  | Outcome | Normalized within 2 months post-discontinuation. |
| 2007 (Long, Wofford et al. 2007) | Drug | Labetalol 400 mg/day PO later increased to 600 mg/day. |
|  | Demographics | 51 y/o African American M |
|  | Phenotype | Hepatocellular |
|  | Latency | 90 D |
|  | Liver enzymes | TB 24 mg/dL, DB 18 mg, ALT, 4026 IU/L, AST, 5753 IU/L, INR 2.0, R = NR |
|  | Histology | Hepatitis with bridging necrosis with no areas of collagenation consistent with necrosis as the result of an acute process. |
|  | Outcome | Patient eventually developed sub-fulminant hepatic failure requiring liver transplantation. |
| **Centrally acting anti-hypertensive** | | |
| 2014 (Kashkooli, Baraty et al. 2014) | Drug | Alpha-Methyldopa 1500 mg/day, 750 mg/day |
|  | Demographics | 34 y/o 2-month post-partum F |
|  | Phenotype | Hepatocellular |
|  | Latency | 150 D |
|  | Liver enzymes | ALP 275 U/L, GGT 123 U/L, AST 1340 U/L, ALT 1018 U/L, INR 1.2 , R = 11.1 |
|  | Histology | Marked hepatic inflammation with mixed, predominantly lymphocytic inflammatory cell infiltrate with focal bridging necrosis. |
|  | Outcome | Improved at 10 days, almost normalized 7–8 weeks post-discontinuation |
| 2010 (Slim, Ben Salem et al. 2010) | Drug | Alpha-methyldopa, dose NR |
|  | Demographics | 34 y/o 13 weeks pregnant F |
|  | Phenotype | Hepatocellular |
|  | Latency | 28 D |
|  | Liver enzymes | AST 412 IU/L, ALT 685 IU/L, TB 161 μmol/L, DB 142 μmol/L, ALP 301 IU/L, GGT 57, Normal INR, R = 6.83 |
|  | Histology | NR |
|  | Outcome | Normalized within 5 weeks post-discontinuation. |
| 2009 (Ali, Srinivasan et al. 2009) | Drug | Alpha-methyldopa, dose NR |
|  | Demographics | 33 y/o 12 weeks African American pregnant F |
|  | Phenotype | Hepatocellular |
|  | Latency | 42 D |
|  | Liver enzymes | NR |
|  | Histology | NR |
|  | Outcome | Improved 2 weeks post-discontinuation |
| 2006 (Phadnis, Sangay et al. 2006) | Drug | Alpha-methyldopa, dose NR |
|  | Demographics | 40 y/o 7-wks pregnant F |
|  | Phenotype | Hepatocellular |
|  | Latency | 14 D |
|  | Liver enzymes | TB 35 µmol/L, ALP 216 IU/L, GGT 237 U/L, ALT 2511 U/L, R = 34.9 |
|  | Histology | NR |
|  | Outcome | Improved after 1–2 weeks and almost 7–8 weeks post-discontinuation |
| Angiotensin Converting Enzyme Inhibitors and Angiotensin Receptor Blockers | | |
| 2023 (Zhang, Cai et al. 2023) | Drug | Sacubitril/valsartan, 200 mg/day |
|  | Demographics | 90 y/o F |
|  | Phenotype | Hepatocellular |
|  | Latency | 4 D |
|  | Liver enzymes | TB 24.5 µmol/L, DB 17.2 µmol/L, AST 796 U/L, ALT 650 U/L. R = NR |
|  | Histology | NR |
|  | Outcome | Improved after stopping |
| 2021 (Odak, Udongwo et al. 2021) | Drug | Olmesartan, Dose NR |
|  | Demographics | 79 y/o African American M |
|  | Phenotype | Hepatocellular |
|  | Latency | NR |
|  | Liver enzymes | AST 191 U/L, ALT 331 U/L, ALP 98 U/L, TB 9.6 mg/dlL DB 5.1 mg/dL, R = 10.1 |
|  | Histology | Lymphocytic-predominant portal chronic inflammatory infiltrate, with cholestasis, and mild steatosis suggesting an autoimmune hepatitis-like pattern. No cirrhosis or iron deposition was identified. No florid duct lesion or granuloma suggesting primary biliary cirrhosis. |
|  | Outcome | LFT’s as well as GI symptoms improved after a month of Olmesartan discontinuation |
| 2021 (Diogo, Monteiro et al. 2021) | Drug | Losartan 50 mg/day. Patient had h/o DILI 4 years prior with unclear trigger at that time (likely Losartan in retrospect) |
|  | Demographics | 66 y/o Caucasian M |
|  | Phenotype | Hepatocellular |
|  | Latency | 21 D |
|  | Liver enzymes | AST 2522 U/L, ALT 3603 U/L, ALP 184 U/L, GGT 375 U/L, INR 1.65, TB 5.28 mg/dL, DB 3.23 mg/dL, R = 47.1 |
|  | Histology | NR |
|  | Outcome | Initially did not improve with Losartan discontinuation so at Day 6, prednisone was started with gradual improvement. |
| 2020 (Hermida Perez, Izquierdo Romero et al. 2020) | Drug | Candesartan, dose NR |
|  | Demographics | 55 y/o M |
|  | Phenotype | Cholestatic |
|  | Latency | 21 D |
|  | Liver enzymes | ALT 130 U/L, ALP 1,033 U/L, GGT 842 U/L, AST 97 U/L, TB 4.7 mg/dL, DB 3.8 mg/dL, R = 0.38 |
|  | Histology | Mixed inflammatory infiltrate with isolated eosinophils and pronounced ductular proliferation with no involvement of the epithelium compatible with toxicity induced damage. |
|  | Outcome | Normalized by 6 months after discontinuation. |
| 2019 (Kogachi, Treyzon et al. 2019) | Drug | Olmesartan, dose NR |
|  | Demographics | 59 y/o M |
|  | Phenotype | Hepatocellular |
|  | Latency | 920 D |
|  | Liver enzymes | Peak ALT 324 U/L, AST 162 U/L 7 months after the onset of symptoms, R = NR |
|  | Histology | Portal inflammation with scattered eosinophils and scattered aggregates of foamy macrophages in the lobules with concerns for liver injury in the setting of a malabsorptive syndrome secondary to olmesartan-associated sprue-like enteropathy |
|  | Outcome | GI symptoms and LFT’s normalized within 3 weeks of cessation |
| 2017 (Park, Yun et al. 2017) | Drug | Fimasartan, 30 mg/day ,60 mg/day |
|  | Demographics | 73 y/o M |
|  | Phenotype | Hepatocellular |
|  | Latency | 300 D |
|  | Liver enzymes | AST 233 U/L, ALT 424 U/L, ALP 112 U/L, GGT 118 U/L, TB 1.22 mg/dL, INR 1.09, R = 11.4 |
|  | Histology | Hepatocellular necrosis seen in zones 2 and 3, with spared periportal hepatocytes in zone suggesting toxic hepatitis. |
|  | Outcome | Normalized 3 months post-discontinuation. |
| 2017 (Lammel-Lindemann, Flores-Villalba et al. 2018) | Drug | Candesartan, 8 mg/day, 16 mg/day |
|  | Demographics | 28 y/o M |
|  | Phenotype | Hepatocellular |
|  | Latency | 7 D |
|  | Liver enzymes | AST 197 IU/L, ALT 578 IU/L, ALP 60 IU/L, TB 1.0 mg/dl, R = 11.6 |
|  | Histology | NR |
|  | Outcome | Improved 2 weeks post-discontinuation, normalized in 4 weeks. |
| 2017 (Forner, Kulai et al. 2017) | Drug | Ramipril, dose NR |
|  | Demographics | 67 y/o M |
|  | Phenotype | Cholestatic |
|  | Latency | 35 D |
|  | Liver enzymes | AST 47 U/L, ALT 46 U/L, ALP 470 U/L, TB 13.2 mg/dL, INR 1.4, R = 0.26 |
|  | Histology | Minimal portal-based inflammation and no interface or lobular hepatitis, no fibrosis with overall picture compatible with medication-induced cholestasis. |
|  | Outcome | Started to improve 6 weeks post-discontinuation. |
| 2017 (Barge, Ziol et al. 2017) | Drug | Olmesartan, 40 mg/day |
|  | Demographics | 61 y/o M |
|  | Phenotype | Hepatocellular |
|  | Latency | 810 D |
|  | Liver enzymes | AST 223, ALT 328, ALP 131, T Bili 0.8 mg/dl, INR 1.11, R = 7.5 |
|  | Histology | Extensive septal fibrosis with lymphocytic portal inflammation and marked interface hepatitis without steatohepatitis |
|  | Outcome | Normalized 3 months post-discontinuation |
| 2014 (Crepin, Godet et al. 2014) | Drug | Aliskiren, 150 mg/day |
|  | Demographics | 61 y/o F |
|  | Phenotype | Hepatocellular |
|  | Latency | 30 D |
|  | Liver enzymes | AST 1631 IU/L, ALT 987 IU/L, ALP 466 IU/L, GGT 820 IU/L, R = 6.4 |
|  | Histology | NR |
|  | Outcome | Improved within a week and normalized in few weeks post-disconsolation. |
| 2013 (Douros, Kauffmann et al. 2013) | Drug | Ramipril, 10 mg/day |
|  | Demographics | 40 y/o M |
|  | Phenotype | 1^st^—Mixed, 2^nd^—Hepatocellular |
|  | Latency | 300 D after first exposure, with 1–2 days of re-exposure |
|  | Liver enzymes | 1^st^ exposure: ALT 317U/L, ALP 364U/L, TB 11.1 mg/dL, R = 2.6  2^nd^ exposure: ALT 1555U/L, ALP 523U/L, TB 1.33 mg/dL, R = 8.92 |
|  | Histology | 2^nd^ event--Parenchymal damage and inflammation with no signs of steatosis. |
|  | Outcome | Improved within few days stopping. |
| 2010 (Zalawadiya, Sethi et al. 2010) | Drug | Lisinopril, 10 mg/day |
|  | Demographics | 30 y/o F |
|  | Phenotype | Hepatocellular |
|  | Latency | 240 D. Various ACEI’s in past, including Lisinopril, without an issue |
|  | Liver enzymes | ALT 1638 U/L, AST 1088 U/L, ALP 175 U/L, TB 12.2 mg/dL, DB 9.5 mg/dL, R = 28.1 |
|  | Histology | Moderate chronic inflammatory cell infiltrates in the portal area, predominantly composed of lymphocytes with mild-to-moderate interface hepatitis. No centrilobular necrosis or steatosis; most compatible with drug-induced hepatotoxicity |
|  | Outcome | Improved 1–2 weeks and normalized by 2 months post-discontinuation. |
| 2010 (da Silva, Alves et al. 2010) | Drug | Enalapril, 10 mg/day for 2.5 year, increased to 20 mg/day 3 months prior to presentation. |
|  | Demographics | 40 y/o white M |
|  | Phenotype | Cholestatic |
|  | Latency | 900 D before 1^st^ presentation, within few days after 2^nd^ and 3^rd^ exposure. |
|  | Liver enzymes | ALT 370 U/L, AST 859 U/L, ALP 668 U/L,GGT 1210 U/L ,TB 16.4 mg/dL, R = 1.66 |
|  | Histology | Periportal confluent necrosis, sinusoidal deposition of fibrin, focal biliary damage and intense ductal metaplasia with macro- and micro-vesicular steatosis associated with neutrophil infiltration and Mallory bodies, occasionally with satellitosis. These alterations were greatest in zone 1 but extended to zone 2 and, more rarely, to zone 3. |
|  | Outcome | Improved within 4–6 weeks post-discontinuation but worsened within few days of restarting (positive rechallenge) |
| 2003 (Bas, Erkan et al. 2003) | Drug | Enalapril, 10 mg/day |
|  | Demographics | 7 y/o girl |
|  | Phenotype | Mixed, Cholestatic |
|  | Latency | 1450 D |
|  | Liver enzymes | AST 1417 IU/L, ALT 1318 IU/L, ALP 727 IU/L, GGT 633 IU/L, TB 1.9mg/dL, DB 1.15 mg/dL,  R = 5.85 |
|  | Histology | Minimal biliary stasis, cholestatic parenchymal changes, moderate inflammatory reaction and slight fibrosis. |
|  | Outcome | Started to Improve one-week post-discontinuation. Patient also treated with  prednisolone for minimal change disease. |
| 2003 (Macias, Campos et al. 2003) | Drug | Enalapril, 10 mg/day |
|  | Demographics | 58 y/o M |
|  | Phenotype | Cholestatic |
|  | Latency | 720 D |
|  | Liver enzymes | AST 286 U/L, ALT 133 U/L, ALP 382 U/L, GGT 215 U/L, TB 10 mg/dL, R = 1.04 |
|  | Histology | Ductopenia and cholestasis with hepatocyte necrosis in centrilobular zones, probably because of hepatotoxicity related to drugs. |
|  | Outcome | Improved 40 days post-discontinuation. |
| 2003 (Yeung, Wong et al. 2003) | Drug | Ramipril, dose NR |
|  | Demographics | Case series of 3 middle aged Males |
|  | Phenotype | Cholestatic |
|  | Latency | ~ 42 D |
|  | Liver enzymes | TB peaked at 15.5 and at 5 mg/dL, ALP peaked at 957 and 507 U/L while serum aminotransferase levels were mildly elevated, R = NR |
|  | Histology | **Case 1**: Marked cholestasis, portal tract expansion, and marked portal and septal fibrosis in a pattern of early biliary cirrhosis. Portal tracts contained a mild to moderate inflammatory infiltrate composed largely of lymphocytes and neutrophils with occasional eosinophils. A few of the original bile ducts showed extensive necrosis of the epithelium, although the total duct number was not definitely decreased. There was prominent ductular proliferation, and many of these ductules and adjacent zone 1 hepatocytes were undergoing necrosis. This process was accompanied by liberated bile in the interstitium and focal collections of foamy or bile-stained macrophages. There were numerous canalicular bile plugs in zone 3.  **Case 2**:  cholestasis with bile duct necrosis, consistent with drug-induced injury  **Case 3**: No biopsy performed. |
|  | Outcome | Cholestasis improved in 1 patient 6 weeks post-discontinuation but prolonged for 14 months in the other, in whom biliary cirrhosis was present on biopsy.  Third patient recovered shortly post-discontinuation. |
| 2003 (Kiykim, Altintas et al. 2003) | Drug | Valsartan, dose NR |
|  | Demographics | 52 y/o F |
|  | Phenotype | Cholestatic |
|  | Latency | 30 D |
|  | Liver enzymes | ALT 780 U/L, AST 1292 U/L, ALP 1840 U/L, GGT 945 U/L, Peak TB 7.8 mg/dL, DB 6.9 mg/dL, R = 1.27 |
|  | Histology | NR |
|  | Outcome | Improved within 2 weeks and normalized within 3 months post-discontinuation. |
| **Diuretics** | | |
| 2021 (Malezieux, Meunier et al. 2021) | Drug | Hydrochlorothiazide, 25 mg/day |
|  | Demographics | 86 y/o F |
|  | Phenotype | Mixed |
|  | Latency | 28 D |
|  | Liver enzymes | AST 263 U/L, ALT 633 U/L, ALP 419 U/L, TB 4.2 mg/dL, DB 3.5mg/dL, R = 4.5 |
|  | Histology | Portal inflammation associated with biliary dystrophies, focal cholangitis, lobular canalicular cholestasis lesions, few eosinophilic polynuclear cells suggestive of DILI. |
|  | Outcome | Normalized 2–3 weeks after discontinuation. |
| 2004 (Arinzon, Alexander et al. 2004) | Drug | Hydrochlorothiazide, 25 mg/day |
|  | Demographics | 72 y/o F |
|  | Phenotype | Mixed |
|  | Latency | 6 D |
|  | Liver enzymes | ALT 237 U/L, AST 145/L, ALP 146 U/L, GGT 363 U/L, R = 3.9 |
|  | Histology | NR |
|  | Outcome | Normalized 3 days post-discontinuation |
| **Vasodilators** | | |
| 2023 (de Oliveira, Kumar et al. 2023) | Drug | Hydralazine, dose NR |
|  | Demographics | 65 y/o F |
|  | Phenotype | Hepatocellular |
|  | Latency | 60 D |
|  | Liver enzymes | ALT 2065 U/L, AST 2055 U/L, TB 15.2 mg/dL, R = NR |
|  | Histology | Extensive hepatocyte necrosis and collapse affecting approximately 90% of the hepatic parenchyma, with accompanying congestion, cholestasis, and acute and chronic inflammation. |
|  | Outcome | Deceased donor liver transplant 5 days after presentation (65 days after drug initiation). |
| 2021 (Bhardwaj, Bhardwaj et al. 2021) | Drug | Hydralazine initially at 150 mg/day, then 300 mg/day 2 weeks later. |
|  | Demographics | 54 y/o F |
|  | Phenotype | Mixed |
|  | Latency | 3 D after 300 mg/day |
|  | Liver enzymes | AST 170 U/L, ALT 155 U/L, TB normal, ALP normal, R = 3.1 |
|  | Histology | Mild inflammation of the portal tracts without significant interface activity, duct injury or ductular reaction, mild steatosis without ballooned hepatocytes, Mallory hyaline, or cholestasis, scattered lobular inflammation without hepatocellular injury in the hepatic parenchyma. |
|  | Outcome | Normalized in 3 weeks |
| 2018 (Sharma, Foge et al. 2018) | Drug | Hydralazine, 150 mg/day |
|  | Demographics | 74 y/o M |
|  | Phenotype | Cholestatic |
|  | Latency | 60 D |
|  | Liver enzymes | ALT 132 U/L, AST 262 U/L, ALP 444 U/L, TB 4.0 mg/dL, R =0.89 |
|  | Histology | NR |
|  | Outcome | Improved few days post-discontinuation, almost to normal after a month. |
| 2016 (Harati, Rahmani et al. 2016) | Drug | Hydralazine 300 mg/day |
|  | Demographics | 75 y/o African American F |
|  | Phenotype | Mixed |
|  | Latency | 70 D |
|  | Liver enzymes | ALT 242 U/L, AST 439 U/L, ALP 166 U/L , TB 22.0 mg/dL, DB 15.7 mg/L, INR 1.23, R = 4.37 |
|  | Histology | Extensive lobular hepatitis, significant necrosis, severe parenchymal collapse, and mixed inflammatory cells infiltrate and trichrome and reticulin staining revealed no significant portal fibrosis with overall picture suggestive of DILI. |
|  | Outcome | Improved after 8 days and normalized by 5 weeks post-discontinuation (was discharged on prednisone) |
| 2009 (Hassan, Hammad et al. 2009) | Drug | Hydralazine 225 mg/day |
|  | Demographics | 63 y/o African American M |
|  | Phenotype | Cholestatic |
|  | Latency | 150 D |
|  | Liver enzymes | NR |
|  | Histology | NR |
|  | Outcome | Improved after a week, normalized 4 weeks post-discontinuation. |
| Abbreviations: AFLP: Acute fatty liver of pregnancy; ALF: Acute Liver Failure; ALP: Alkaline phosphatase; ALT: alanine aminotransferase; AST: Aspartate transaminase; CHOL: Cholestatic; DILI: Drug induced liver injury; DB: Direct bilirubin; GGT: gamma-glutamyl transferase; HC: Hepatocellular; HELLP: Hemolysis, elevated liver enzymes and low platelets syndrome; IB: indirect bilirubin; INR: international normalized ratio; LFT’s: liver function tests; mg/dL: Milligrams per deciliter; mg: milligrams; NR: not reported; POD: post-operative day; TB: Total bilirubin; y/o: year old; U/L: units per liter; y: year | | |
| Only English Articles were included 1 abstract, rest full case reports, with ACEI/ARB’s effect seems to be idiosyncratic. | | |

**Table S2** and Vignette—Severe hepatocellular injury during lisinopril therapy.

| Date | ALT (U/L) | AST (U/L) | Alk P (U/L) | Bilirubin (mg/dL) | INR | Albumin (g/dL) | *R* ratio |
| --- | --- | --- | --- | --- | --- | --- | --- |
| 25NOV2009 | 141 | 254 | 137 | 6.9 | N/A | 3.6 | 10.3 |
| 30NOV2009 | 56 | 94 | 105 | 20.8 | 1.40 | N/A | 5.3 |
| 14DEC2009 | 34 | 131 | 111 | 41.2 | 2.40 | 3.4 | 0.7 |
| 21DEC2009 | 830 | 1570 | 73 | 10.4 | 1.30 | N/A | 26.0 |
| 09FEB2010 | 22 | 20 | 94 | 1.2 | N/A | 4.4 | 0.5 |

A 34-year-old male with newly diagnosed hypertension was treated with lisinopril/hydrochlorothiazide (50 mg daily) starting November 2, 2009, and later switched to amlodipine (5 mg daily) on November 16, 2009. He developed jaundice, dark urine, itching, fatigue, and chills two weeks later on November 24, 2009. He was admitted to the hospital on November 25 with marked elevations in serum aminotransferase levels and bilirubin, consistent with severe hepatocellular injury. After extensive workup, no other causes were identified. His condition worsened, leading to renal insufficiency and necessitating liver transplantation on December 20, 2009. Histology confirmed sub-fulminant hepatic failure, likely drug-induced. Post-transplant recovery was favorable. *HLA* genotyping for all relevant alleles was negative.

**Key Points**

**Medications:** Lisinopril with hydrochlorothiazide, Amlodipine

**Pattern:** Hepatocellular

**Severity:** Severe (requiring liver transplantation)

**Latency:** 3 weeks after lisinopril; 1 week after amlodipine

**Recovery:** Post-liver transplantation

**Figure S2** Injury from lisinopril superimposed on cirrhosis due to steatohepatitis. (A) There is extensive perisinusoidal fibrosis isolating small regenerative nodules. (Masson trichrome, 40x). (B) At high magnification, there is canalicular cholestasis and multiple Mallory-Denk bodies. (H&E, 400x).

**Table S3** and Vignette: Severe hepatocellular injury associated with hydralazine therapy.

| Date | ALT (U/L) | AST (U/L) | Alk P (U/L) | Bilirubin (mg/dL) | INR | Albumin (g/dL) | *R* ratio |
| --- | --- | --- | --- | --- | --- | --- | --- |
| 07JAN2011 | 1005 | 1410 | 239 | 19.8 | N/A | 2.6 | 11.5 |
| 13JAN2011 | 586 | 891 | 211 | 18.7 | 2.00 | Not Done | 4.9 |
| 28JAN2011 | 212 | 203 | 176 | 20.2 | 2.70 | Not Done | 2.1 |
| 03FEB2011 | 130 | 116 | 130 | 17.9 | 2.90 | Not Done | 1.8 |
| 15AUG2011 | 21 | 16 | 334 | 0.4 | Not Done | 4.2 | 0.1 |

A 42-year-old African American male with no prior liver disease developed severe hepatocellular injury 4 months and 1 week after starting hydralazine (50 mg BID) for hypertension. He discontinued the drug on December 1, 2010, due to headaches. Short time after that, he experienced flu-like symptoms, followed by jaundice on December 20, 2010. His initial labs on January 7, 2011, showed ALT >1000 U/L, AST >1400 U/L, bilirubin >19 mg/dL, and alkaline phosphatase >200 U/L. A liver biopsy was taken on January 13, 2011, revealed significant necrosis, severe collapse, and a mixed inflammatory infiltrate. Hydralazine was identified as the likely cause. Despite prednisone treatment, his bilirubin and INR remained elevated. The patient underwent liver transplantation on February 3, 2011, and recovered well post-transplant. The patient was positive for HLA *HLA-B*35:01 and HLA-C*04:0.*

**Key Points**

- **Medications:** Hydralazine, Doxazosin
- **Pattern:** Hepatocellular
- **Severity:** Severe (requiring liver transplantation)
- **Latency:** 127 days after starting hydralazine
- **Recovery:** Post-liver transplantation

**Figure S3** There is extensive hepatocellular necrosis associated with marked ductular reaction and residual chronic inflammation. There were only rare residual hepatocytes in the biopsy. (H&E, 100x).

**Table S4** and Vignette—Cholestatic liver injury associated with Lisinopril therapy leading to death.

| Date | ALT (U/L) | AST (U/L) | Alk P (U/L) | Bilirubin (mg/dL) | INR | Albumin (g/dL) | *R* ratio |
| --- | --- | --- | --- | --- | --- | --- | --- |
| 30MAR2021 | 111 | 154 | 710 | 5.7 | N/A | Not Done | 0.3 |
| 01APR2021 | 107 | 141 | 744 | 6.2 | 2.0 | Not Done | 0.3 |
| 27AUG2021 | 33 | 60 | 338 | 5.7 | 1.7 | Not Done | 0.2 |
| 22SEP2021 | 38 | 62 | 562 | 7.1 | 2.0 | Not Done | 0.2 |
| 04FEB2022 | 18 | 55 | 265 | 24.4 | 2.9 | 2.9 | 0.1 |

A 65-year-old African American female with a history of hypertension, diabetes mellitus, and chronic depression developed cholestatic hepatitis after taking lisinopril for an unknown duration. She developed jaundice and itching on March 25, 2021. Initial labs on March 30, 2021, showed elevated liver enzymes: ALT 111 U/L, AST 154 U/L, alkaline phosphatase 710 U/L, and total bilirubin 5.7 mg/dL. Despite stopping lisinopril on September 8, 2021, her liver function continued to decline, imaging confirmed cirrhosis and portal hypertension. The patient was evaluated for a liver transplant, but she was not a good candidate due to frailty and psychosocial issues. She was placed on home hospice and died on February 14, 2022. The patient was positive for HLA *HLA-B*35:01, HLA-C*04:0, HLA-DQB1*05:01 and HLA-DQA1*01:01.*

**Key Points**

- **Medications:** Lisinopril
- **Pattern:** Cholestatic
- **Severity:** Severe (leading to death)
- **Latency:** Onset after long-term use of lisinopril
- **Recovery:** No recovery; patient died

**Table S5** and Vignette—Cholestatic liver injury associated with hydralazine therapy.

| Date | ALT (U/L) | AST (U/L) | Alk P (U/L) | Bilirubin (mg/dL) | INR | Albumin (g/dL) | *R* ratio |
| --- | --- | --- | --- | --- | --- | --- | --- |
| 12MAY2015 | 70 | 52 | 212 | 1.2 | N/A | Not Done | 0.6 |
| 28JUL2015 | 231 | 256 | 288 | 5.6 | 2.5 | 2.5 | 1.4 |
| 29JUL2015 | 164 | 122 | 318 | 7.0 | 2.3 | 2.3 | 0.9 |
| 30JUL2015 | 199 | 218 | 575 | 7.0 | 1.6 | 2.3 | 0.6 |
| 11AUG2015 | 47 | 31 | 248 | 1.6 | 1.3 | 1.3 | 0.3 |

A 72-year-old male with a history of a kidney transplant, chronic kidney disease (stage III), congestive heart failure, and hypertension developed cholestatic liver injury shortly after starting hydralazine for hypertension. The patient started hydralazine on July 13, 2015, and developed symptoms of jaundice and fatigue by July 27, 2015. Laboratory tests on July 28, 2015, showed elevated liver enzymes: ALT 231 U/L, AST 256 U/L, alkaline phosphatase 288 U/L, and total bilirubin 5.6 mg/dL. Despite stopping hydralazine on July 29, 2015, the patient’s liver function initially worsened before gradually improving. A liver biopsy was planned but was canceled due to clinical improvement. The patient was discharged on August 11, 2015, with liver enzyme levels near normal. HLA genotyping for all relevant alleles was negative.

**Key Points**

- **Medications:** Hydralazine
- **Pattern:** Cholestatic
- **Severity:** Moderate (hospitalized but recovered)
- **Latency:** 14 days after starting hydralazine
- **Recovery:** Gradual improvement after discontinuation

**Table S6** Approximate relative risks of DILI due to anti-hypertensive drugs in the drug-induced liver injury network study, 2004–2022. [Numbers of cases enrolled relative to numbers of persons for which the drugs were prescribed in 2021]

| Drug | Number of cases in DILIN | Number of persons drug prescribed in the USA in 2021 | Number of cases/1 million persons prescribed |
| --- | --- | --- | --- |
| Methyldopa* | 13 | < 158,742 | >81.8* |
| Hydralazine | 11 | 1,624,406 | 6.77 |
| Lisinopril +/– HCTZ | 6 | 23,502,316 | 0.255 |
| Verapamil | 3 | 580,801 | 5.164 |
| Benazepril+/– HCTZ or amlodipine | 2 | 1,989,412 | 1.006 |
| Atenolol | 1 | 3,384,297 | 0.296 |
| Carvedilol | 1 | 5,039,099 | 0.198 |
| Enalapril | 1 | 190,679 | 5.24 |
| Irbesartan | 1 | 744,799 | 1.344 |
| Losartan | 1 | 13,269,279 | 0.075 |
| Labetalol | 1 | 507,522 | 1.969 |
| Quinapril* | 1 | <158,742 | >6.29* |
|  |  |  |  |
| Hydrochlorothiazide | 0 | 9,655,980 | 0 |
| Metoprolol | 0 | 15,535,072 | 0 |
| Amlodipine | 0 | 17,734,288 | 0 |
| Furosemide | 0 | 6.473,988 | 0 |
| Spironolactone | 0 | 3.146,100 | 0 |
| Diltiazem | 0 | 2,091,547 | 0 |
| Clonidine | 0 | 1,835,614 | 0 |
| Nifedipine | 0 | 1,237,401 | 0 |
| Olmesartan | 0 | 1,173,921 | 0 |
| Triamterene | 0 | 1,392,875 | 0 |
| Chlorthalidone | 0 | 1,281,201 | 0 |
| Propranolol | 0 | 2,008,047 | 0 |
| Timolol | 0 | 872,159 | 0 |
| Torsemide | 0 | 530,362 | 0 |
| Irbesartan | 0 | 744,799 | 0 |
| Telmisartan | 0 | 481,997 | 0 |
| Doxazosin | 0 | 593,551 | 0 |
| Prazosin | 0 | 512,048 | 0 |
| Terazosin | 0 | 492,399 | 0 |

Prescription data are from ClinCalc.com/Drug Stats for 2021, the 300 most prescribed drugs in the USA, latest data available. *Data for alpha-methyl DOPA and Quinapril are not exact because they were not among the 300 most prescribed drugs.

**Table S7** Conditional analysis on *HLA B*35:01* in the overall cohort of 44 anti-HTN DILI cases and DILI from other drugs (non-anti-HTN DILI).

| Drug | Allele | Carriage frequency (anti-HTN DILI cases) | Carriage frequency (non-anti-HTN DILI cases) | OR (95% CI) | *P*-value |
| --- | --- | --- | --- | --- | --- |
| Overall (44 *vs.* 1447) | *C*04:01* | 0.59 | 0.24 | 1.2 (0.6-2.1) | 0.61 |
|  | *DQA1*01:01* | 0.48 | 0.26 | 1.6 (0.9-2.7) | 0.12 |
|  | *DQB1*05:01* | 0.43 | 0.23 | 1.3 (0.7-2.3) | 0.35 |

Results were from the logistic regression model, adjusted for PC1, PC2, PC3, and diabetes, and conditioned on *B*35:01*.

**Table S8** Haplotypes involving the Top 4 alleles in patients with anti-HTN DILI and DILI ascribed to other drugs (non-anti-HTN DILI)

| Ethnicity | Allele | Haplotype frequency (anti-HTN DILI cases) | Haplotype frequency (non-anti-HTN DILI cases) | *P*-value |
| --- | --- | --- | --- | --- |
| Overall (44 *vs.* 1447) | *B*35:01-C*04:01* | 0.21 | 0.05 | 3×10^-11^ |
|  | *B*35:01-DQB1*05:01* | 0.14 | 0.02 | 9×10^-13^ |
|  | *B*35:01-DQA1*01:01* | 0.14 | 0.03 | 2×10^-12^ |
|  | *B*35:01-C*04:01-DQA1*01:01* | 0.13 | 0.03 | 3×10^-12^ |
|  | *B*35:01-C*04:01-DQB1*05:01* | 0.14 | 0.02 | 8×10^-12^ |
|  | *B*35:01-DQA1*01:01-DQB1*05:01* | 0.14 | 0.02 | 1×10^-13^ |
|  | *B*35:01-C*04:01-DQA1*01:01-DQB1*05:01* | 0.13 | 0.02 | 4×10^-13^ |
| NHW (24 vs. 1058) | *B*35:01-C*04:01* | 0.23 | 0.05 | 3×10^-7^ |
|  | *B*35:01-DQB1*05:01* | 0.10 | 0.03 | 7×10^-5^ |
|  | *B*35:01-DQA1*01:01* | 0.10 | 0.03 | 0.0001 |
|  | *B*35:01-C*04:01-DQA1*01:01* | 0.13 | 0.03 | 8×10^-5^ |
|  | *B*35:01-C*04:01-DQB1*05:01* | 0.10 | 0.03 | 6×10^-5^ |
|  | *B*35:01-DQA1*01:01-DQB1*05:01* | 0.10 | 0.03 | 6×10^-5^ |
|  | *B*35:01-C*04:01-DQA1*01:01-DQB1*05:01* | 0.10 | 0.03 | 5×10^-5^ |
| NHB (16 vs. 181) | *B*35:01-C*04:01* | 0.13 | 0.03 | 0.006 |
|  | *B*35:01-DQB1*05:01* | 0.16 | 0.02 | 5×10^-10^ |
|  | *B*35:01-DQA1*01:01* | 0.16 | 0.01 | 2×10^-14^ |
|  | *B*35:01-C*04:01-DQA1*01:01* | 0.13 | 0.008 | – |
|  | *B*35:01-C*04:01-DQB1*05:01* | 0.13 | 0.01 | 0.002 |
|  | *B*35:01-DQA1*01:01-DQB1*05:01* | 0.16 | 0.01 | 2×10^-6^ |
|  | *B*35:01-C*04:01-DQA1*01:01-DQB1*05:01* | 0.13 | 0.003 | – |
| HISPANIC (4 vs. 134) | *B*35:01-C*04:01* | 0.25 | 0.04 | 0.006 |
|  | *B*35:01-DQB1*05:01* | 0.25 | 0 | – |
|  | *B*35:01-DQA1*01:01* | 0.25 | 0.007 | – |
|  | *B*35:01-C*04:01-DQA1*01:01* | 0.25 | 0.01 | 2x10^-11^ |
|  | *B*35:01-C*04:01-DQB1*05:01* | 0.25 | 0.004 | – |
|  | *B*35:01-DQA1*01:01-DQB1*05:01* | 0 | – | – |
|  | *B*35:01-C*04:01-DQA1*01:01-DQB1*05:01* | 0.25 | 0 | – |

Overall analysis uses PC1, PC2, PC3, and diabetes covariates with the haplo.score default model. Other analyses use the haplo.score default model without covariates due to sample size.

**Table 9** Summary statistics of the most significant *HLA* alleles comparing antihypertensive-DILI cases to dbGAP population controls across ethnicities. The table also shows the effect of the alleles in each drug group.

| *HLA* Allele | CF cases | CF Pop Controls | OR (95% CI) | *P* |  |
| --- | --- | --- | --- | --- | --- |
| Overall (*n*=44; Population Controls *n*=5456*)^#^* | | | | | |
| *B*35:01* | 0.50 | 0.13 | 5.3 (3.1–8.9) | 3×10^-8*^ |  |
| *C*04:01* | 0.59 | 0.27 | 2.8 (1.7–4.4) | 5×10^-5*^ |  |
| *DQA1*01:01* | 0.48 | 0.18 | 3.5 (2.1–5.6) | 7×10^-6*^ |  |
| *DQB1*05:01* | 0.43 | 0.23 | 2.1 (1.2–3.4) | 0.007^*^ |  |
| ACEI+ARB (*n*=14*;* Population Controls *n*=1736)^^^ | | | | | |
| *B*35:01* | 0.43 | 0.12 | 4.2 (1.4–10.9) | 0.006 |  |
| *C*04:01* | 0.43 | 0.24 | 2.2 (0.8–5.5) | 0.08 |  |
| *DQA1*01:01* | 0.36 | 0.19 | 2.5 (0.8–6.4) | 0.05 |  |
| *DQB1*05:01* | 0.29 | 0.22 | 1.7 (0.5–4.5) | 0.37 |  |
| Methyldopa (*n*=13; Population Controls *n*=1612)^ | | | | | |
| *B*35:01* | 0.69 | 0.14 | 7.0 (2.7–16.9) | 4.8×10^-5*^ |  |
| *C*04:01* | 0.85 | 0.30 | 4.3 (1.8–10.0) | 0.0005^*^ |  |
| *DQA1*01:01* | 0.62 | 0.10 | 4.3 91.6–10.4) | 0.002^*^ |  |
| *DQB1*05:01* | 0.54 | 0.24 | 2.4 (0.9–6.1) | 0.07 |  |
| Hydralazine (*n*=11; Population Controls *n*=5456)^ | | | | | |
| *B*35:01* | 0.27 | 0.14 | 2.1 (0.4–7.1) | 0.20 |  |
| *C*04:01* | 0.36 | 0.28 | 1.2 (0.3–3.7) | 0.77 |  |
| *DQA1*01:01* | 0.27 | 0.19 | 1.5 (0.3–5.0) | 0.47 |  |
| *DQB1*05:01* | 0.27 | 0.25 | 1.0 (0.2–3.5) | >0.99 |  |

^#^ Results from logistic regression. ^^^ Results were from Fisher’s exact test. Case:Control Ratio 1:12 for population controls, 1:5 for Non-Anti-HTN DILI. * *Q* < 0.05. Abbreviations: OR- odds ratio; CI- confidence interval;

**Table S10** List of Causal drugs and percentage of *HLA-B*35:01* positive carriers

| Drug | Non-carrier | Carrier | Carrier Freq |
| --- | --- | --- | --- |
| Atenolol | 1 (4.5%) | 0 (0.0%) | 0% |
| Benazepril | 1 (4.5%) | 1 (4.5%) | 50% |
| Carvedilol | 0 (0.0%) | 1 (4.5%) | 100% |
| Enalapril | 1 (4.5%) | 0 (0.0%) | 0% |
| Hydralazine | 8 (36.4%) | 3 (13.6%) | 30% |
| Irbesartan | 0 (0.0%) | 1 (4.5%) | 100% |
| Labetalol | 0 (0.0%) | 1 (4.5%) | 100% |
| Lisinopril | 3 (13.6%) | 3 (13.6%) | 50% |
| Losartan | 2 (9.1%) | 0 (0.0%) | 0% |
| Methyldopa | 4 (18.2%) | 9 (40.9%) | 69% |
| Quinapril | 1 (4.5%) | 0 (0.0%) | 0% |
| Valsartan | 0 (0.0%) | 1 (4.5%) | 0% |
| Verapamil | 1 (4.5%) | 2 (9.1%) | 67% |
|  |  |  |  |

**Table S11** Effect of the most significant *HLA* risk allele within major ethnicities comparing methyldopa cases to DILI ascribed to non-anti-HTN drugs (non-anti-HTN DILI)

| Ethnicity | Allele | Carriage frequency (methyldopa DILI cases) | Carriage frequency (non-anti-HTN DILI cases) | OR (95% CI) | *P*-value |
| --- | --- | --- | --- | --- | --- |
| Overall (13 *vs.* 1447) | *B*35:01* | 0.69 | 0.11 | 9.0 (3.5–21.8) | 8×10-6* |
|  | *C*04:01* | 0.85 | 0.24 | 5.7 (2.4–13.5) | 4×10-5* |
|  | *A*68:02* | 0.23 | 0.03 | 12.9 (3.1–40.6) | 0.001* |
|  | *DRB1*15:03* | 0.23 | 0.03 | 7.3 (1.4–25.2) | 0.01 |
|  | *DQA1*01:01* | 0.62 | 0.26 | 2.7 (1.0–6.6) | 0.02 |
|  | *B*53:01* | 0.23 | 0.05 | 5.0 (0.9–17.0) | 0.03 |
|  | *DQB1*05:01* | 0.54 | 0.23 | 2.7 (0.9–6.7) | 0.03 |
|  | *DRB1*08:04* | 0.15 | 0.02 | 7.7 (0.8–33.3) | 0.03 |
| NHW (4 *vs.* 1058) | *B*35:01* | 1 | 0.11 | 16.7 (3.1–90.9) | 0.001** |
|  | *C*04:01* | 1 | 0.22 | 7.5 (1.4–40.7) | 0.01 |
|  | *A*68:02* | 0 | 0.009 | – | – |
|  | *DRB1*15:03* | 0 | 0.001 | – | – |
|  | *DQA1*01:01* | 0.75 | 0.26 | 3.7 (0.6–19.4) | 0.09 |
|  | *B*53:01* | 0 | 0.006 | – | – |
|  | *DQB1*05:01* | 0.75 | 0.22 | 4.5 (0.7–23.2) | 0.06 |
|  | *DRB1*08:04* | 0 | 0.004 | – | – |
| NHB (8 *vs.* 181) | *B*35:01* | 0.50 | 0.09 | 6.3 (1.3–23.7) | 0.01 |
|  | *C*04:01* | 0.75 | 0.40 | 2.6 (0.8–8.3) | 0.07 |
|  | *A*68:02* | 0.25 | 0.11 | 3.5 (0.6–14.3) | 0.08 |
|  | *DRB1*15:03* | 0.38 | 0.24 | 1.6 (0.3–6.2) | 0.44 |
|  | *DQA1*01:01* | 0.50 | 0.27 | 2.0 (0.5–7.0) | 0.27 |
|  | *B*53:01* | 0.38 | 0.31 | 1.1 (0.2–4.2) | 0.74 |
|  | *DQB1*05:01* | 0.38 | 0.30 | 1.2 (0.2–4.7) | 0.73 |
|  | *DRB1*08:04* | 0.25 | 0.12 | 2.2 (0.2–10.6) | 0.27 |
| Hispanic (1 *vs.* 134) | *B*35:01* | 1 | 0.13 | – | – |
|  | *C*04:01* | 1 | 0.25 | – | – |
|  | *A*68:02* | 1 | 0.06 | – | – |
|  | *DRB1*15:03* | 0 | 0.03 | – | – |
|  | *DQA1*01:01* | 1 | 0.27 | – | – |
|  | *B*53:01* | 0 | 0.04 | – | – |
|  | *DQB1*05:01* | 1 | 0.25 | – | – |
|  | *DRB1*08:04* | 0 | 0.04 | – | – |

* *Q* < 0.01; ** *Q* < 0.05; “–“ means allele frequency or sample size was too small for a reliable test

**Table S12** Conditional analysis for Methyldopa DILI on *B*35:* and DILI ascribed to other drugs (non-anti-HTN DILI)

| Drug | Allele | Carriage frequency (methyldopa DILI cases) | Carriage frequency (non-anti-HTN DILI cases) | OR (95% CI) | *P*-value |
| --- | --- | --- | --- | --- | --- |
| Overall (13 *vs.* 1447) | *C*04:01* | 0.85 | 0.24 | 2.6 (0.8–7.2) | 0.10 |
|  | *A*68:02* | 0.23 | 0.03 | 10.7 (3.2–30.3) | 0.0007* |
|  | *DRB1*15:03* | 0.23 | 0.03 | 11.4 (2.5–40.6) | 0.004* |
|  | *DQA1*01:01* | 0.62 | 0.26 | 1.6 (0.6–4.0) | 0.32 |
|  | *B*53:01* | 0.23 | 0.05 | 5.0 (1.2–14.1) | 0.03 |
|  | *DQB1*05:01* | 0.54 | 0.23 | 1.5 (0.5–3.8) | 0.41 |
|  | *DRB1*08:04* | 0.15 | 0.02 | 10.0 (1.6–39.4) | 0.02 |

* *Q* < 0.05

**Table S13** Effect of autoimmune hepatitis-related alleles by causal anti-HTN drugs versis DILI ascribed to non-antihypertensive drugs (non-anti-HTN DILI)

| Drug | Allele | Carriage frequency (anti-HTN DILI cases) | Carriage frequency (non-anti-HTN DILI cases) | OR (95% CI) | *P*-value |
| --- | --- | --- | --- | --- | --- |
| *Overall (N=44)#* | DRB1*03:01 | 0.20 | 0.22 | 0.9 (0.4–1.7) | 0.81 |
|  | *DRB1*04:01* | 0.18 | 0.10 | 2.1 (0.9–4.2) | 0.07 |
|  | *DR3* | 0.25 | 0.23 | 0.9 (0.5–1.7) | 0.79 |
|  | *DR4* | 0.27 | 0.26 | 1.3 (0.7–2.3) | 0.39 |
| *ACEI+ARB (N=14)^* | DRB1*03:01 | 0.29 | 0.22 | 1.3 (0.3–3.7) | 0.56 |
|  | *DRB1*04:01* | 0.21 | 0.10 | 2.1 (0.4–6.9) | 0.20 |
|  | *DR3* | 0.29 | 0.23 | 1.1 (0.3–3.7) | 0.77 |
|  | *DR4* | 0.36 | 0.26 | 1.3 (0.4–3.5) | 0.59 |
| *Methyldopa (N=13)^* | DRB1*03:01 | 0.23 | 0.22 | 1.0 (0.2–3.3) | >0.99 |
|  | *DRB1*04:01* | 0.31 | 0.10 | 3. 1(0.8–9.4) | 0.06 |
|  | *DR3* | 0.23 | 0.23 | 0.9 (0.2–3.0) | >0.99 |
|  | *DR4* | 0.31 | 0.26 | 1.1 (0.3–3.17) | 0.78 |
| *Hydralazine (N=11)^* | DRB1*03:01 | 0.18 | 0.22 | 0.8 (0.1–3.2) | >0.99 |
|  | *DRB1*04:01* | 0.09 | 0.10 | – | – |
|  | DR3 | 0.36 | 0.23 | 1.5 (0.4–4.7) | 0.51 |
|  | *DR4* | 0.27 | 0.26 | 1.3 (0.3–4.0) | 0.55 |

^#^Results were from the logistic regression model, adjusted for PC1, PC2, PC3, and diabetes. ^^^ Results were from Fisher’s exact test. **Q* < 0.05. ***Q* < 0.15 Abbreviations: OR- odds ratio; CI- confidence interval;

**Table 14** Association between ANA or SMA antibody carriers and *HLA-B*35:01*, *HLA-DRB1*04:01*, and both carriers

| Allele | ANA/SMA positive  *HLA* carriage freq | ANA/SMA negative  *HLA* carriage freq | OR 95%CI | *P* |
| --- | --- | --- | --- | --- |
| *B*35:01 or DRB1*04:01* | 18/23 = 0.78 | 7/17 = 0.41 | 5.8 (1.3–30.7) | 0.02 |
| *B*35:01* | 15/23 = 0.65 | 6/17 = 0.35 | 4.4 (1.1–22.0) | 0.04 |
| *DRB1*04:01* | 4/23 = 0.17 | 4/17 = 0.24 | 0.6 (0.1–3.3) | 0.53 |

Note: some cases are missing ANA and/or SMA data; these are excluded; *P*= *P*-value from logistic regression with PC1 to PC2 as covariates; “ANA/SMA Positive” means a positive lab test for ANA, or SMA, “ANA/SMA Negative” means a negative lab test for ANA or SMA,

**Table S15** Effect of *HLA-B*53:01* within major ethnicities comparing all anti-HTN DILI cases to DILI ascribed to non-antihypertensive drugs (non-anti-HTN DILI)

| Ethnicity | Allele | Carriage frequency (anti-HTN DILI cases) | Carriage frequency (non-anti-HTN DILI) | OR (95% CI) | *P*-value |
| --- | --- | --- | --- | --- | --- |
| Overall (44 *vs.* 1447) | *B*53:01* | 0.14 | 0.05 | 1.0 (0.4–2.4) | 0.95 |
| NHW (24 *vs.* 1058) | *B*53:01* | 0 | 0.006 | – | – |
| NHB (16 *vs.* 181) | *B*53:01* | 0.38 | 0.31 | 1.1 (0.4–2.9) | 0.81 |
| HISPANICS (4 *vs.* 134) | *B*53:01* | 0 | 0.04 | – | – |
